# Construction of a high-density SNP-based genetic map and identification of fruit-related QTLs and candidate genes in peach [*Prunus persica* (L.) Batsch]

**DOI:** 10.1186/s12870-020-02557-3

**Published:** 2020-09-23

**Authors:** Pei Shi, Ze Xu, Shaoyu Zhang, Xianju Wang, Xiaofei Ma, Jicheng Zheng, Libo Xing, Dong Zhang, Juanjuan Ma, Mingyu Han, Caiping Zhao

**Affiliations:** College of horticulture, Northwest A&F University, Yangling, 712100 Shaanxi China

**Keywords:** SNP-based map, Peach, SLAF, QTL, Fruit quality-related traits

## Abstract

**Background:**

High-density genetic mapping is a valuable tool for mapping loci that control specific traits for perennial fruit trees. Peach is an economically important fruit tree and a model Rosaceae species for genomic and genetic research. In peach, even though many molecular markers, genetic maps and QTL mappings have been reported, further research on the improvement of marker numbers, map densities, QTL accuracy and candidate gene identification is still warranted.

**Results:**

A high-density single nucleotide polymorphism (SNP)-based peach linkage map was constructed using specific locus amplified fragment sequencing (SLAF-seq). This genetic map consisted of 7998 SLAF markers, spanning 1098.79 cM with an average distance of 0.17 cM between adjacent markers. A total of 40 QTLs and 885 annotated candidate genes were detected for 10 fruit-related traits, including fruit weight (FW), fruit diameter (FD), percentage of red skin colour (PSC), eating quality (EQ), fruit flavour (FV), red in flesh (RF), red around pit (RP), adherence to pit (AP), fruit development period (FDP) and fruit fibre content (FFC). Eighteen QTLs for soluble solid content (SSC) were identified along LGs 1, 4, 5, and 6 in 2015 and 2016, and 540 genes were annotated in QTL intervals. Thirty-two QTLs for fruit acidity content (FA) were detected on LG1, and 2, 4, 5, 6, and 1232 candidate genes were identified. The expression profiles of 2 candidate genes for SSC and 4 for FA were analysed in parents and their offspring.

**Conclusions:**

We constructed a high-density genetic map in peach based on SLAF-seq, which may contribute to the identification of important agronomic trait loci. Ninety QTLs for 12 fruit-related traits were identified, most of which overlapped with previous reports, and some new QTLs were obtained. A large number of candidate genes for fruit-related traits were screened and identified. These results may improve our understanding of the genetic control of fruit quality traits and provide useful information in marker-assisted selection for fruit quality in peach breeding programmes.

## Background

Peach [*Prunus persica* (L.) Batsch] is well-known as a delicious and healthy summer fruit in the temperate regions of the world with a total production of approximately 2.47 million tonnes worldwide in 2017 (http://www.fao.org/faostat/en/#data). Peach is an important perennial fruit tree species with few chromosomes (2n = 2x = 16), a relatively small genome (~ 230 Mbp), and a short juvenile period (2–4 years) [1]. These traits make peach a model plant in Rosaceae fruit trees for genetic map construction, important agronomic trait location, and target candidate gene identification.

Fruit quality is a complex trait involving fruit appearance, texture, taste, flavour and so on. Infante et al. (2008) overviewed the fruit quality evaluation through physical, chemical and sensorial parameters, and the inheritance and molecular breeding of the main fruit quality traits in peach [2]. Most of these quality traits are quantitatively inherited and controlled by multiple loci. The construction of genetic maps and quantitative trait loci (QTL) analysis are effective strategies for the identification of candidate genes associated with fruit quality traits [3].

The first peach map was reported by Chaparro et al. using intraspecific F2 progeny, including 83 random amplified polymorphic DNA (RAPD) markers, 1 isozyme and 4 morphological markers [4]. Subsequently, with the development of molecular marker technology, numerous peach genetic linkage maps were constructed [5–9].

Sosinski et al. detected 12 QTLs for soluble solids, pH, cold tolerance, maturation date, and fruit size [5]. Dirlewanger et al. identified 32 QTLs related to sugar and acid contents, and epistasis was observed between QTLs [10]. The molecular markers tightly linked to several Mendelian agronomic characteristics were also detected. Freestone (F) was located in G4, low acid (D) and peach (G) in G5, and pollen sterility (Ps), flat (S), and aborting fruit (Af) in G6 [6]. A cluster of QTLs for fruit weight (FW), juice total soluble solids (SSC), and juice titratable acidity (FA) was found at a distal position on G4 close to the endoPG CAPS marker, and QTLs for maturity date (MD) were also found on G4 [7]. Fresnedo-Ramírez et al. identified 5 QTLs that accounted for up to ~ 29% of the phenotypic variation of fruit equatorial diameter (FD) and up to ~ 17% of fresh weight (FW) [11]. Hernández Mora et al. identified 47 QTLs for the seven most important agronomic traits of peach by the integrated analysis of 18 families from different European breeding programmes [12].

Significant QTL effects were detected on linkage group 4 for fruit mealiness (M) and flesh bleeding (FBL) and on linkage group 5 for flesh browning (FBr) [8]. Sánchez et al. identified QTLs for volatile compounds in peach fruit, and QTL mapping showed clustering of volatile QTLs included in the same volatile modules. A unique locus at the top of LG4 controlled the monoterpene module [13]. Eduardo et al. identified three major QTLs for nonanal, linalool, and p-menth-1-en-9-al in linkage group 4. The genes encoding two putative terpene synthases and one lipoxygenase (Lox) might be involved in the biosynthesis of linalool and p-menth-1-en-9-al and nonanal, respectively [14]. Bielenberg et al. detected ten QTLs for chilling requirement (CR) and nineteen QTLs for bloom date (BD) [15]. Two stony hard (SH) phenotype-related QTLs were found in linkage group LG6. Three genes (Prupe.6G150900.1, Prupe.6G147600.1 and Prupe.6G156500.1) were identified as candidates for the SH trait [9].

In recent years, expression QTLs (eQTLs) was used to identify the candidate genes located inside QTL regions for the fruit quality traits. For example, García-Gómez et al. (2019) identified QTLs linked to fruit quality traits of apricot, and obtained a candidate gene, MYB transcription factor, for skin colour in LG3, and three candidate genes for the SSC in LG4 through gene expression analysis [16]. Carrasco-Valenzuela et al. (2019) had developed an integrative analysis involving conventional QTLs, eQTLs (expression QTLs), and transcriptome profiling to identify candidate genes involved in peach fruit softening rate, and indicated that auxin biosynthetic related genes triggered fast softening in melting peach fruit [17]. Although many molecular marker, genetic map and QTL analyses have been reported, the accuracy of QTL mapping needs to be improved, and concise information on the number and position of the genes determining the inheritance of a given trait is lacking [12].

In this study, we constructed a high-density genetic map using SNP markers which be developed through specific-locus amplified fragment sequencing (SLAF-seq), and then we identified stable QTLs associated with 12 fruit quality traits (including fruit weight, fruit diameter, fruit skin colour, red in flesh, red around pit, adherence to pit, fruit development period, fruit fibre content, fruit flavor, eating quality, soluble solid content and fruit acidity content) for two years in an F1 population. Subsequently, we screened the candidate genes located inside these stable QTL regions and verified them through gene expression analysis using qPCR. Our results will be beneficial for understanding the genetic basis for the formation of peach fruit quality, thus providing a theoretical basis for improving fruit quality via MAS and / or genetic manipulation.

## Results

### Fruit-related trait phenotypic identification

**Fig. 1:**
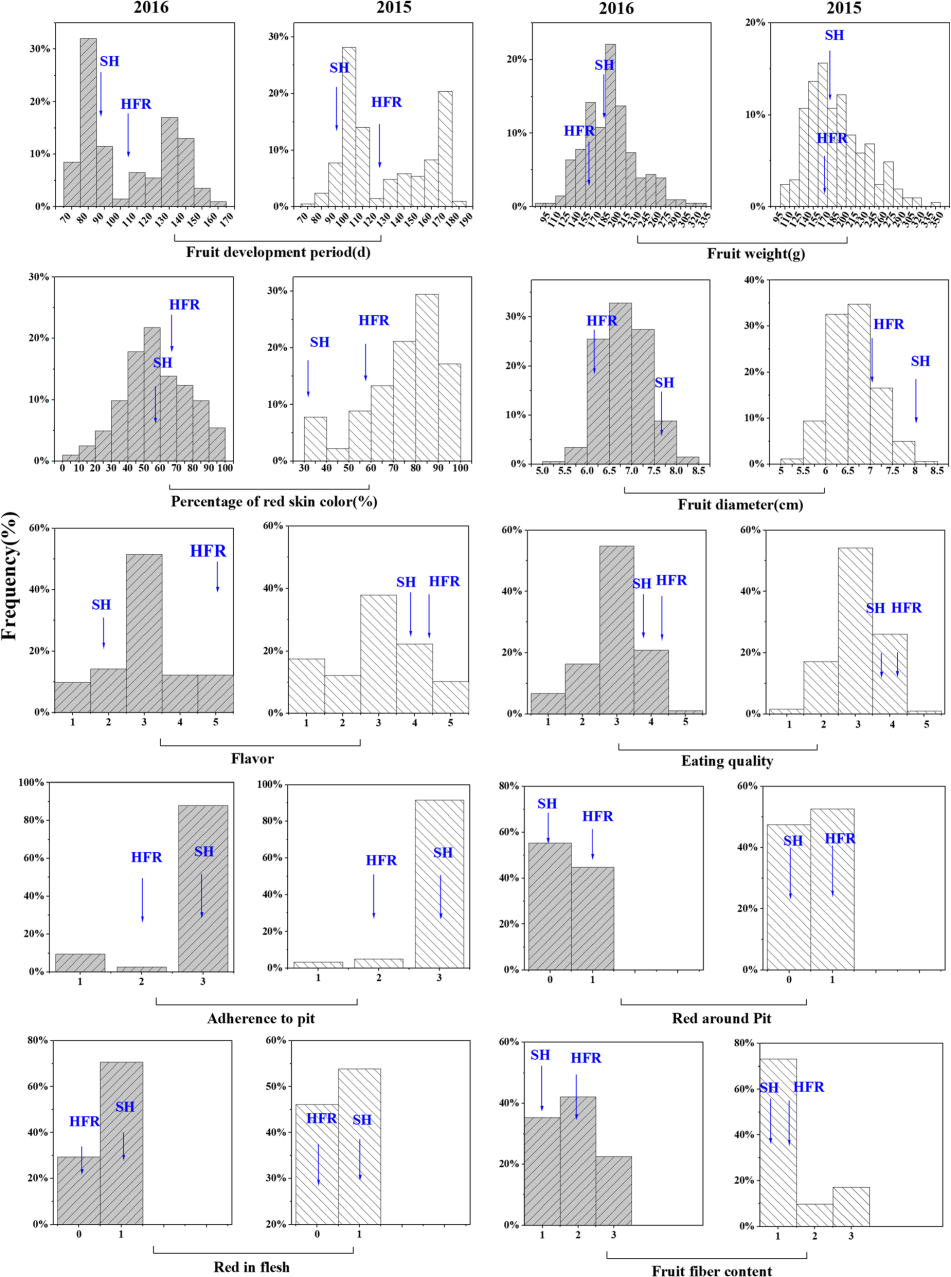
Distribution of phenotypes for fruit quality traits (including fruit development period, fruit weight, fruit diameter, percentage of red skin colour, red in flesh, red around pit, adherence to pit, eating quality), fruit flavour and fruit fibre content measured in 2015 and 2016 on the F1 progeny. Notes: Fruit flavour (1 = sour, 2 = sour-sweet, 3 = water sweet, 4 = sweet, 5 = rich sweet); eating quality (1 = extremely poor, 2 = poor, 3 = fair, 4 = good, 5 = excellent); adherence to pit (1 = freestone, 2 = semi-freestone, 3 = clingstone); red around pit (0 = no red, 1 = red); fruit fibre content (1 = few, 2 = intermediate, 3 = many); red in flesh (0 = no red overlay, 1 = red overlay). The values of the parents ‘Shahong’ (SH) and ‘Hongfurong’ (HFR) are indicated by arrows

**Fig. 2:**
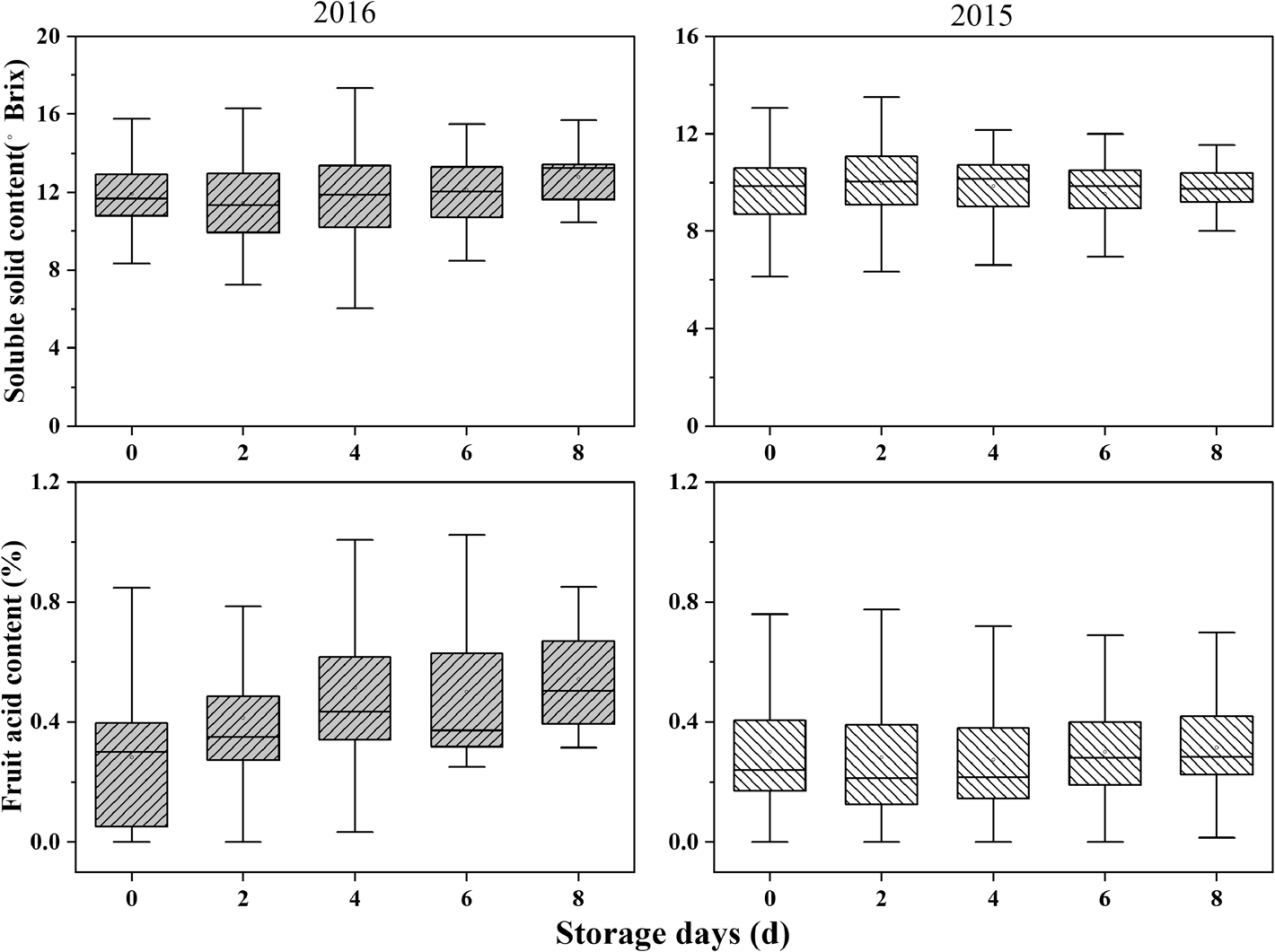
Boxplot distributions of soluble solid content and fruit acidity content in ‘Shahong’ × ‘Hongfurong’ F1 progeny measured during storage at 25 °C for the years 2015 and 2016

Twelve fruit-related traits from 202 individuals of the F1 population were analysed in 2015 and 2016. The group of quantitative traits included fruit development period (FDP), fruit weight (FW), fruit diameter (FD), percentage of red skin colour (PSC), eating quality (EQ), fruit flavour (FV), soluble solid content (SSC), and fruit acidity content (FA). FW, FD, PSC, FV and EQ had normal distributions in progeny, and FDP showed a significant non-normal distribution (Fig. 1). Therefore, FDP was treated as a qualitative trait in the subsequent analysis. SSC and FA of parents and F_1_ progenies were measured during fruit storage. SSC showed a slight upward trend during storage in both 2015 and 2016, while FA increased in 2016 and remained stable in 2015. In addition, the SSC and FA in 2016 were higher than those in 2015 (Fig. 2). The group of qualitative traits included red in flesh (RF), red around pit (RP), adherence to pit (AP), fruit fibre content (FFC) and FDP. QTLs for these qualitative traits were detected using the Kruskal–Wallis test.

### High-density genetic linkage map construction for peach

A total of 7998 out of 8037 high-quality polymorphic SLAF markers were distributed into eight linkage groups (LGs) according to their locations in the *Prunus persica* genome (Fig. S1). The integrated genetic map spanned 1098.79 cM, with an average distance of 0.17 cM between the adjacent markers. LG1 was the largest LG containing 1699 markers, covering 143.89 cM, with an average distance of only 0.08 cM and a maximum gap of only 2.67 cM being observed between the adjacent markers. LG8 had the fewest markers of 386 and spanned a length of 113.03 cM, with an average distance of 0.29 cM and a maximum gap of 5.86 cM being observed between the adjacent markers (Table 1). In addition, the collinearity of mapping markers between the physical and genetic maps was determined with Spearman correlations. A high level of collinearity was found, represented by scores of less than or equal to 1.0 (Fig. S2).

**Table 1:** Distribution of mapped markers among eight linkage groups in peach

Linkage Group ID	Total Marker	Total Distance (cM)	Average Distance (cM)	Max Gap
Chr1	1699	143.89	0.08	2.67
Chr2	1397	107.65	0.08	4.51
Chr3	1025	184.81	0.18	7.12
Chr4	1462	167.07	0.11	11.70
Chr5	506	148.45	0.29	15.45
Chr6	740	116.71	0.16	5.30
Chr7	783	117.17	0.15	5.44
Chr8	386	113.03	0.29	5.86
Total	7998	1098.79	0.17	15.45

### QTLs identified for fruit-related traits

The QTL analysis of 12 fruit-related traits was performed by using MapQTL6.0 software with the above SLAF-based high density linkage map. For FW, FD, PSC, EQ and FV with normal distributions, a total of 21 QTLs were detected. Four QTLs were identified for FW, of which two were located on LG 4 (7 and 6.4%), the third was located on LG 5 (5.3%), and the fourth was located on LG 6 (5.8%). Four QTLs were identified for FD, two of which were located on LG 4 (5.9 and 10.3%), and the other two were located on LG 5 (6.8 and 6.0%). Five QTLs were identified for PSC, two of which were located on LG 4 (7 and 7.5%), and the remaining 3 QTLs were located on LG 1 (6.4%), LG 3 (4.9%), and LG 6 (6.6%). Two QTLs for EQ were located on LG 1 (7 and 6.8%). Six QTLs were identified for FV, of which two were located on LG 1 (7.7 and 8.1%), two were located on LG5 (13.9 and 5.3%), and two were located on LG3 (10.4%) and LG4 (6.7%) (Table 2).

**Table 2:** Identified QTLs of 10 fruit-related traits

I. QTLs of quantitative traits using interval mapping and multiple QTL model method											
Trait	Year	LG	Cofactor Marker	QTL	Genetic Position			Physical Position		LOD	explained variations (%)
					Start (cM)	Final (cM)	Interval (cM)	Start (bp)	Final (bp)		
Fruit weight (FW)	2015	4	Marker415082371	qP-FW4.1^1^	97.3	98.289	0.99	15,082,371	16,710,388	2.81	7.0
	2016	4	Marker46320002	qP-FW4.2^2^	45.027	45.421	0.39	6,316,366	6,320,223	2.47	6.4
	2016	5	Marker57632555	qP-FW5.1^2^	55.908	56.194	0.29	7,318,892	7,816,084	2.2	5.3
	2016	6	Marker628159465	qP-FW6.1^2^	108.055	108.055	0.00	28,159,465	28,159,465	2.54	5.8
Fruit diameter (FD)	2015	4	Marker415082371	qP-FD4.1^1^	97.3	98.289	0.99	15,082,371	16,710,388	3.84	10.3
	2016	4	Marker46320002	qP-FD4.2^2^	45.027	45.027	0.00	6,320,002	6,320,002	2.59	5.9
	2015	5	Marker57664342	qP-FD5.1^1^	55.908	55.908	0.00	7,664,342	7,664,342	2.16	6.8
	2016	5	Marker57632555	qP-FD5.2^2^	55.908	56.194	0.29	7,318,892	7,816,084	2.67	6.0
Percentage of red skin color (PSC)	2016	1	Marker121499106	qP-PSC1.1^2^	56.814	57.179	0.37	21,487,305	21,513,105	2.8	6.4
	2016	3	Marker310648307	qP-PSC3.1^1^	108.701	108.709	0.08	10,648,307	10,838,614	2.06	4.9
	2015	4	Marker419804890	qP-PSC4.1^1^	121.898	121.915	0.02	19,804,890	19,842,357	2.58	7.0
	2016	4	Marker428152553	qP-PSC4.2^2^	149.991	150.074	0.08	28,152,553	28,561,890	3.27	7.5
	2015	6	Marker626821847	qP-PSC6.1^1^	99.526	99.537	0.01	26,821,847	26,822,849	2.52	6.6
Eating quality (EQ)	2016	1	Marker121482495	qP-EQ 1.1^2^	55.794	56.344	0.55	21,332,095	21,482,979	2.55	7.0
	2015	1	Marker126704889	qP-EQ 1.2^1^	77.286	77.458	0.17	26,561,825	26,750,141	2.56	6.8
Flavor (FV)	2016	1	Marker127158729	qP-FV1.1^2^	78.031	80.198	2.17	26,694,116	27,334,450	3.36	7.7
	2016	1	Marker128065739	qP-FV1.2^2^	83.271	86.952	3.68	27,653,862	28,166,262	3.52	8.1
	2016	3	Marker33722468	qP-FV3.1^2^	31.417	70.374	38.96	3,128,322	4,476,565	4.56	10.4
	2015	4	Marker417553353	qP-FV4.1^1^	100.574	103.631	3.06	15,270,537	17,635,472	2.99	6.7
	2015	5	Marker5638231	qP-FV5.1^1^	20.72	20.72	0.00	638,231	638,231	2.2	13.9
	2015	5	Marker51499828	qP-FV5.2^1^	42.642	42.897	0.25	1,499,828	1499,831	2.33	5.3
**II**. QTLs of qualitative traits using Kruskal-Wallis method											
Trait	Year	LG	Cofactor marker	QTL	Genetic Position			Physical Position		K*	Signif.
					Start (cM)	Final (cM)	Interval (cM)	Start (bp)	Final (bp)		
Red in flesh (RF)	2016	1	Marker124532043	qP-RF1.1^2^	68.951	72.768	3.82	24,430,498	25,072,844	11.255	****
	2015	1	Marker124908662	qP-RF1.2.^1^	68.826	76.049	7.22	24,397,147	26,479,742	8.896	****
Red around pit (RP)	2016	1	Marker126704889	qP-RP1.1^2^	77.016	77.286	0.27	26,671,055	26,704,889	7.21	**
	2015	1	Marker126704889	qP-RP1.2^1^	77.286	77.286	0.00	26,704,889	26,704,889	6.673	**
	2016	1	Marker126747840	qP-RP1.3^2^	77.458	77.458	0.00	26,747,840	26,750,141	8.585	**
	2015	1	Marker126747840	qP-RP1.4^1^	77.458	77.458	0.00	26,747,840	26,750,141	6.053	**
	2016	1	Marker126757867	qP-RP1.5^2^	77.56	77.56	0.00	26,757,867	26,757,867	12.311	****
	2015	1	Marker126757867	qP-RP1.6^1^	77.56	77.56	0.00	26,757,867	26,757,867	6.595	**
	2016	1	Marker127176678	qP-RP1.7^2^	79.16	79.16	0.00	27,176,678	27,176,678	13.627	****
	2015	1	Marker127176678	qP-RP1.8^1^	79.16	79.16	0.00	27,176,678	27,176,678	6.063	**
Adherence to Pit (AP)	2016	3	Marker37112788	qP-AP3.1^2^	78.489	86.066	7.58	6,258,941	7,861,876	4.788	**
	2015	3	Marker37112788	qP-AP3.2^1^	78.489	86.066	7.58	6,258,941	7,861,876	5.768	**
	2016	7	Marker715748214	qP-AP7.1^2^	44.76	44.76	0.00	15,748,214	15,748,214	11.619	****
	2015	7	Marker715748214	qP-AP7.2^1^	44.76	44.76	0.00	15,748,214	15,748,214	12.766	****
	2015	4	Marker423822119	qP-AP4.1^1^	141.869	142.718	0.85	23,822,117	23,822,119	9.372	***
	2016	4	Marker414000094	qP-AP4.2^2^	76.372	76.372	0.00	14,000,094	14,000,094	7.168	**
	2016	4	Marker418581993	qP-AP4.3^2^	110.133	110.133	0.00	18,581,993	18,581,993	7.073	**
Fruit development period (FDP)	2016	7	Marker72036314	qP-FDP7.1^2^	2.454	2.521	0.07	1,838,911	2,036,314	6.564	**
	2015	7	Marker72036314	qP-FDP7.2^1^	2.454	2.521	0.07	1,838,911	2,036,314	5.472	**
	2016	4	Marker420753150	qP-FDP4.1^2^	126.847	126.847	0.00	20,753,150	20,753,150	11.007	****
	2015	4	Marker45665856	qP-FDP4.2^1^	40.716	40.716	0.00	5,665,842	5,665,856	6.152	**
	2015	4	Marker416420904	qP-FDP4.3^1^	108.949	108.949	0.00	16,420,904	16,420,904	8.099	****
Fruit fiber content (FFC)	2015	2	Marker27416433	qP-FFC2.1^1^	30.062	30.353	0.29	7,416,433	7,501,740	4.244	****
	2016	3	Marker314273878	qP-FFC3.1^2^	124.161	124.346	0.19	14,273,878	14,298,880	3.95	**
	2015	3	Marker316663661	qP-FFC3.2^1^	138	138.039	0.04	16,663,659	16,663,661	10.159	***
	2016	7	Marker76260019	qP-FFC7.1^2^	12.237	12.237	0.00	6,260,019	6,260,019	6.52	**
	2015	7	Marker76260019	qP-FFC7.2^1^	12.237	12.237	0.00	6,260,019	6,260,019	8.318	**

K*: the Kruskal–Wallis test statistic. **: 0.01; ***: 0.001; ****: 0.0001

In total, 19 QTLs were identified for 5 qualitative traits, RF, RP, AP, FDP and FFC (the same QTL in 2 years was considered as one QTL). Two overlapping QTLs for RF were located on LG 1. Four QTLs were identified for RP on LG 1 in both 2015 and 2016. Five QTLs were identified for AP, three of which were located on LG 4, and the other two were located on LG 3 and LG 7. Four QTLs were identified for FDP, of which one was located on LG 7, and the other three were located on LG 4. Four QTLs were identified for FFC, of which two were located on LG 3, and the other two were located on LG 2 and LG 7 (Table 2, Fig. S3). Furthermore, most QTLs were confirmed in both 2015 and 2016 and in the same location (Table 2, Fig. S3). The stability of loci for these qualitative traits in different years confirmed the reliability of QTLs.

**Fig. 3:**
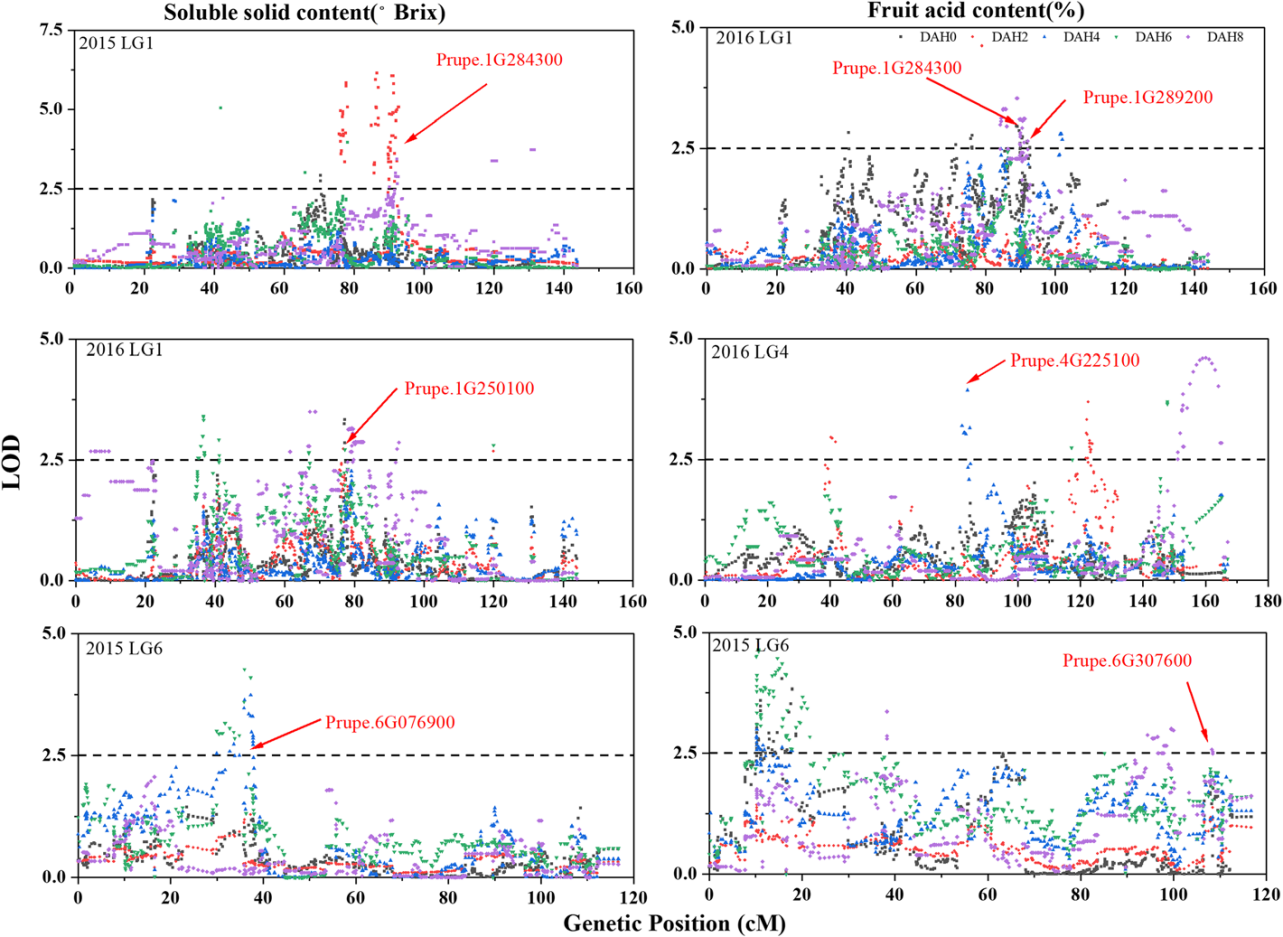
Dynamic QTLs of soluble solid content (SSC) and fruit acid content (FA) during fruit storage. DAH: day after harvest. The locations of the loci for candidate genes associated with SSC and FA are shown by arrows

Dynamic QTLs for SSC and FA based on measured data from the peach fruit storage period were detected in 2015 and 2016. A total of 18 QTLs for SSC were identified along LGs 1, 4, 5, and 6 in 2015 and 2016. Ten QTLs on LG1 were located at 41.102 ~ 42.728 cM (Pp01:13,177,641.. 15,291,518), 66.897 ~ 67.265 cM (Pp01: 24,241,572.. 24,284,070), 75.744 ~ 77.560 cM (Pp01: 25,898,412.. 26,757,867), 91.990 ~ 120.785 cM (Pp01: 29,246,116.. 42,673,647). Two QTLs on LG4 were located at 149.153 ~ 149.934 cM (Pp04: 28,137,742.. 30,186,866). Two QTLs on LG5 were located at 27.451 ~ 30.996 cM (Pp05: 846,656.. 1,094,221). Four QTLs on LG6 were located at 29.965 ~ 37.885 cM (Pp06: 4,716,586.. 5,153,723) (Fig. 3, Fig. S3). Thirty-two QTLs for FA were detected: 3 QTLs were located on LG1, 3 QTLs were on LG2, 9 QTLs were on LG4, 6 QTLs were on LG5, and 11 QTLs were on LG6 (Fig. 3, Fig. S3).

### Potential candidate genes in the QTL interval of fruit-related traits

Potential candidate genes involved in 12 fruit-related traits were investigated according to their QTL intervals with the physical positions on the peach genome. For SSC, 540 annotated candidate genes were identified in the above 18 QTL regions (Table S1). These candidate genes are involved in such processes as the biosynthesis of secondary metabolites, fructose and mannose metabolism, and fatty acid metabolism (Fig. S4).

For FA, 1232 annotated genes were detected in 32 QTL regions (Table S1). These candidate genes are focused on such processes as the biosynthesis of secondary metabolites, protein processing in the endoplasmic reticulum, starch and sucrose metabolism, carbon fixation in photosynthetic organisms, and C5-branched dibasic acid metabolism (Fig. S5). These candidate genes also included such genes as ATP-citrate lyase, the aluminium-activated malate transporter, vacuolar proton ATPase, and the auxin efflux carrier family protein, which may be related to FA. In addition, sucrose transporter 2, UDP-glucose 6-dehydrogenase family protein, 6-phosphogluconolactonase, fructokinase-like 2, sugar transporter protein, fructose-2,6-bisphosphatase and glucose-6-phosphate dehydrogenase, which may be related to the sugar content of the fruit, have been identified for FA (Table S1).

For the remaining 10 fruit-related traits, 885 annotated candidate genes on QTL regions were detected, 130 for FW and FD (FW and FD candidate genes completely overlap), 11 for PSC, 21 for EQ, 186 for RF, 125 for AP, 3 for RP, 6 for FDP, 401 for FV and 2 for FFC (Table S2). Candidate genes for these fruit quality traits were significantly enriched in the secondary metabolic pathway (Fig. S6-S9). In addition, candidate genes for FW are also involved in the biosynthesis of amino acids and protein processing in the endoplasmic reticulum (Fig. S6). Candidate genes for FV are focused on such processes as carbon metabolism, citrate cycle (TCA cycle), and fructose and mannose metabolism (Fig. S7). Candidate genes for EQ are also enriched in such processes as starch and sucrose metabolism (Fig. S8), and RFs are enriched in, for instance, ABC transporters and phenylpropanoid biosynthesis (Fig. S9).

### Expression analysis of selected candidate genes for SSC and FA

**Fig. 4:**
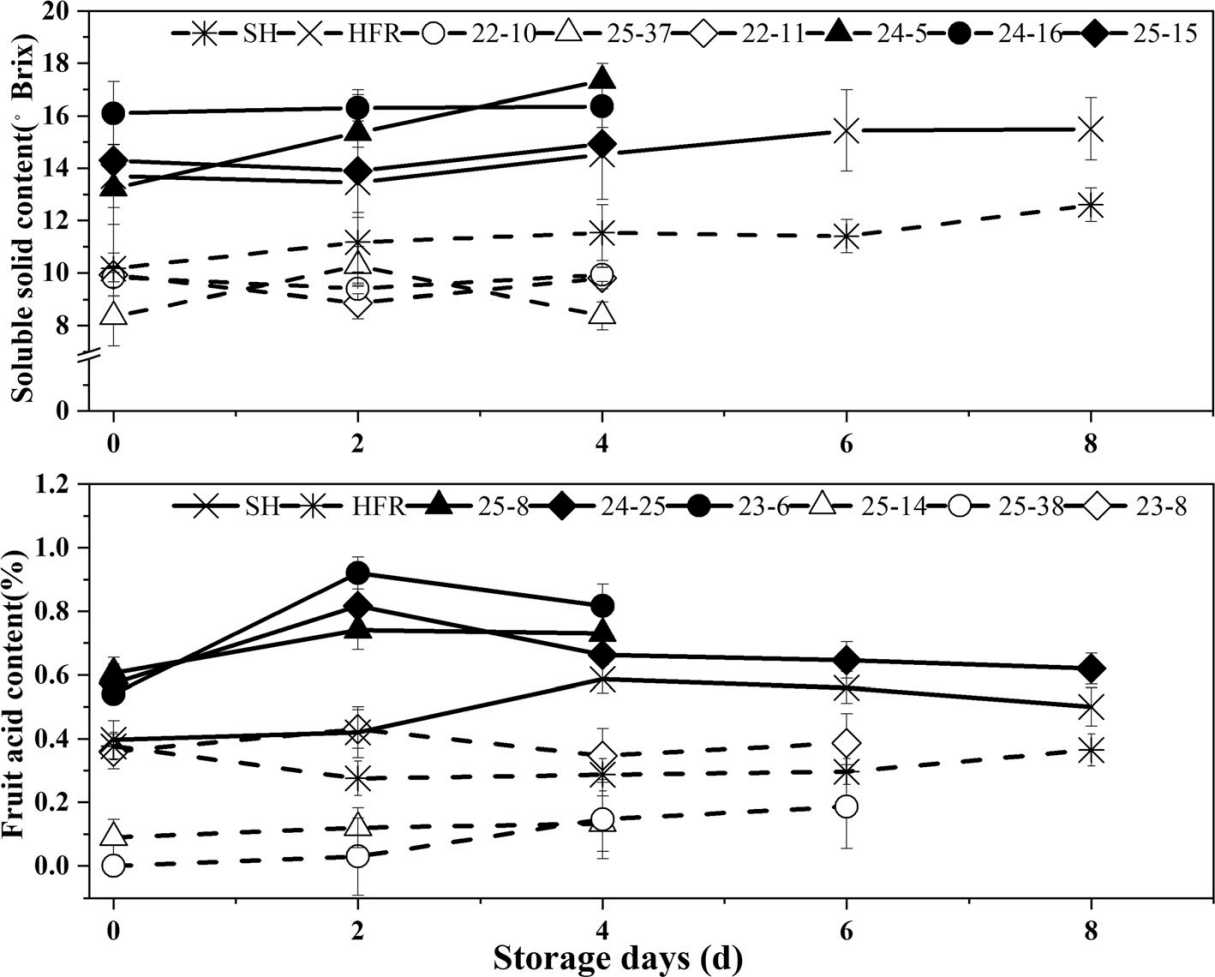
Change in soluble solid content (SSC) and fruit acid content (FA) in fruits of different genotypes during storage

**Fig. 5:**
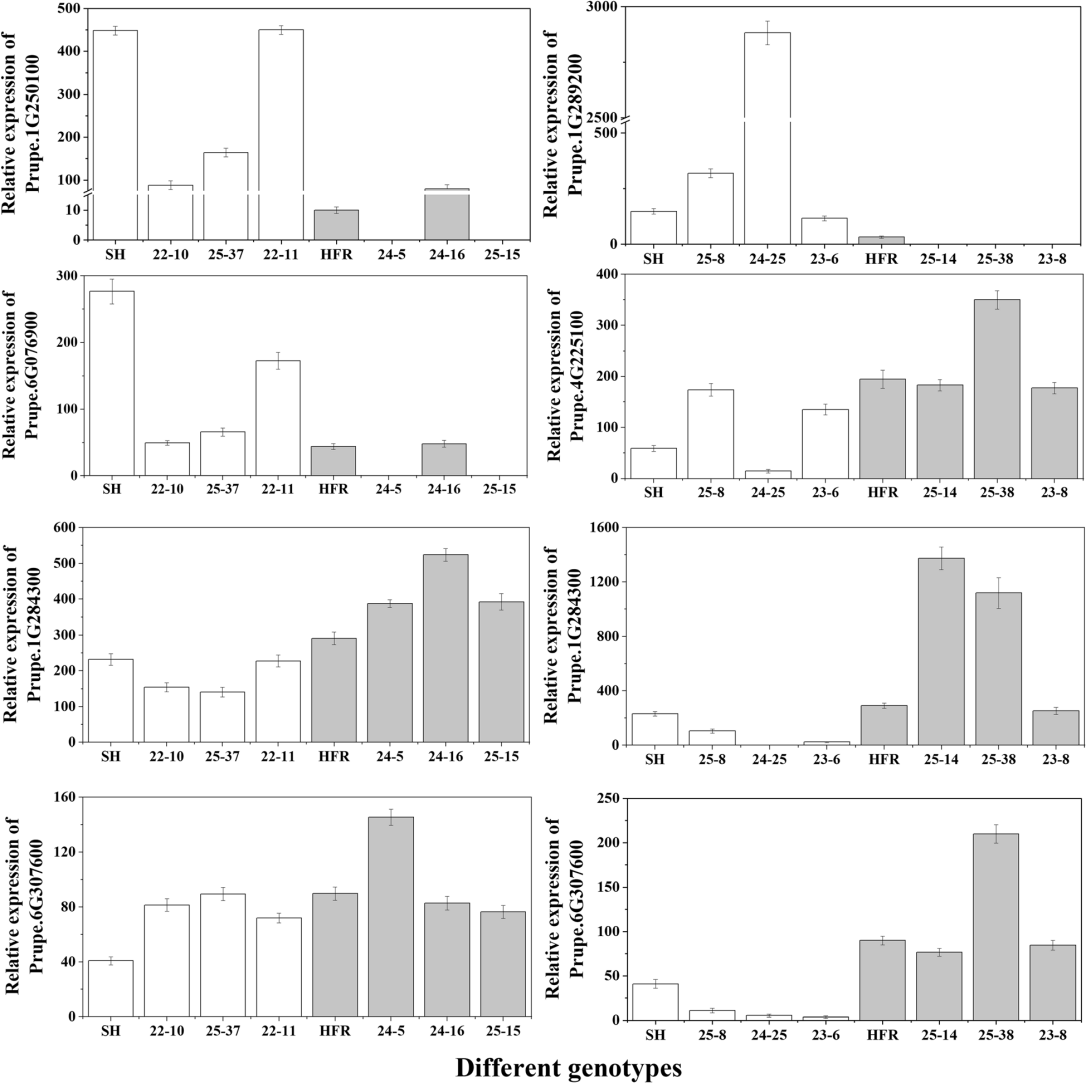
Change in the relative expression of candidate genes in mature fruits

To further confirm the relationship between the candidate genes and fruit SSC and FA, we selected 6 candidate genes, 2 for SSC and 4 for FA, to evaluate their expression in parents and representative offspring. For the SSC trait, HFR, 24–5, 24–16 and 25–15 showed high SSC, while SH, 22–10, 22–11, and 25–37 had low SSC during fruit storage (Fig. 4). qRT–PCR results showed that Prupe.1G250100 and Prupe.6G076900 exhibited significant differences between SH and HFR at the mature stage. The expression levels of Prupe.1G250100 and Prupe.6G076900 in the 6 hybrids were negatively correlated with changes in SSC (Fig. 5). Prupe.1G250100 and Prupe.6G076900 encode ATP-citrate lyase A-2 and O-glycosyl hydrolase family 17 proteins, respectively.

For the FA trait, SH, 23–6, 24–25 and 25–8 showed high FA, while HFR, 25–14, 25–38 and 23–8 had low FA during fruit storage (Fig. 4). The expression of Prupe.1G289200 in SH and offspring with high FA was higher than that in HFR and offspring with low FA (Fig. 5). The expression of Prupe.4G225100, Prupe.1G284300 and Prupe.6G307600 was more highly expressed in low FA fruits than in high FA fruits at the mature stage (Fig. 5). Prupe.1G289200, Prupe.1G284300, Prupe.4G225100 and Prupe.6G307600 encode 6-phosphogluconolactonase, UDP-glucose 6-dehydrogenase, ATPase H+ transporting V0 subunit E (ATPase, V0 complex, subunit E) and glucose-6-phosphate dehydrogenase, respectively. Because Prupe.1G284300 and Prupe.6G307600 are involved in the sugar metabolic pathway, we also detected the expression of Prupe.1G284300 and Prupe.6G307600 in parents and offspring with different SSCs. The results showed that the expression of Prupe.1G284300 was significantly positively correlated with the fruit SSC (Fig. 5).

## Discussion

### High-density SNP genetic map for peach

Molecular markers, such as AFLPs, RAPDs, RFLPs, SSRs and SNPs, have been widely used for genetic map construction and QTL identification in peach [4, 5, 14, 18]. Previously constructed peach linkage maps mainly used AFLPs, RAPDs, RFLPs and SSRs markers; due to the lower availability of these markers, the map density was not high enough and the adjacent marker gaps were larger [4, 5, 18–21]. Recently, a large number of SNP markers were used to construct genetic maps with high-density, and to screen the QTLs of fruit quality related traits [8, 9, 14, 15]. SNP is the most common genetic variation in the plant genome, and it is a highly important genetic marker for constructing a high-density genetic map and completing molecular marker-assisted breeding [22, 23]. Next-generation high-throughput sequencing technologies have recently facilitated the large-scale discovery of genome-wide SNP markers. SLAF-seq is a cost-effective technology for SNP discovery and genotyping and has been applied for genetic map construction and QTL detection in many plant species [24–29]. In addition, the high-density SNP array also was used in high-throughput genotyping, genetic map construction and genome-wide association study of fruit traits [30]. The IPSC 9 K SNP array was commercially available for peach [31]. However, this array was more expensive, and the SNPs on the array were not evenly spaced across the peach genome with adjacent marker gaps up to 1254 kb for some genomic regions [31].

In this study, we constructed a high-density genetic linkage map for peach with 8 linkage groups and 7998 SLAF markers. This genetic map spanned 1098.79 cM with an average distance of 0.17 cM/marker. Dirlewanger et al. (1998) established a peach map covering 712 cM, and the average density between pairs of markers was 4.5 cM [32]. Blenda et al. (2007) published a map including 151 AFLP and 21 SSR markers covering the peach genome of 737 cM with an average marker spacing of 4.7 cM [33]. The Pop-DG intraspecific peach linkage map covered 818.2 cM of the peach genome with an average interval of 4.0 cM between markers [34]. Martínez-García et al. (2013) constructed a peach map with 588 SNP markers and map coverage of 454 cM and an average distance of 0.81 cM/marker site [8]. Nuñez-Lillo et al. (2015) built a linkage map with 1830 SNPs and 7 SSR markers spanning 389.2 cM distributed over eight linkage groups with an average interval of 0.21 cM/marker pair [3]. Guo et al. (2018) published a linkage map with 1310 SNP markers spanning 454.2 cM with an average marker distance of 0.347 cM [9]. Compared with the published linkage maps, the genetic group constructed in our study covered a longer genetic distance and contained a higher marker density. The differences in the length and density of genetic maps may be related to the genetic distance between the parents, the number of markers, and the population size used in different studies. The increased marker number and density could enhance the mapped QTL number as well as the precision [35, 36]. Our experiment results showed that SLAF-based SNP markers were highly effective for constructing high-density genetic maps.

### QTLs identified for fruit-related traits in peach

Fruit quality is a composite trait, and breeding for fruit quality traits is complex due to the polygenic nature of the genetic control of these traits [37]. Mapping QTLs controlling fruit quality in peach has been widely reported [7, 8, 38–41]. Fruit weight (FW) and fruit diameter (FD) are typical quantitative traits controlled by polygenes. The QTLs for FW were linked to LG 6 [10], LG 2, 4, 6 [7], LG 2, 4, 5 [42], LG 1, 4, 6, 7 [43] and LG 1, 2, 3, 5, 6, 7 [44]. FD QTLs were found in linkage groups 1, 2, 3, and 7 [42]. García-Gómez et al. (2019) reported that in ‘G × C’ progeny, the most important QTLs for FW on LG1 and for fruit diameter on LG1 and LG3 [16]. Cao et al. (2016) mapped the association regions for FW on each scaffold by GWAS [45]. We identified QTLs for FW and FD on LG4, 5, and 6. The region of QTL qP-FW4.1^1^ (Pp04: 15,082,371..16,710,388) was close to FW-2010-15 (Pp04:17,625,270) [45] and the FW marker SNP_IGA_410336 (Pp04:10,665,019) [44]; QTL qP-FW4.2^2^ (Pp04: 6,316,366.. 6,320,223) was close to FW-2010-12 (Pp04:8,634,341) [45]; qP-FW5.1^2^ (Pp05: 7,318,892.. 7,816,084) was close to SNP_IGA_574551(Pp05: 6,287,541) [41], and FW-2010-19 (Pp05: 9432987) [45]; qP-FW6.1^2^ (Pp06: 28,159,465.. 28,159,465) was close to FW-2010-21 (Pp06: 20, 400,960) [45]. In addition, our results also showed that most of the QTLs for FW and FD were highly coincident, which may be due to the significant correlation between fruit weight and fruit diameter.

Pigmentation (skin and flesh colour) is an important fruit quality trait in commercial peaches. Verde et al. (2002) detected 2 QTLs for fruit skin colour; one QTL was placed in LG 2, and the other was placed in LG 6 [46]. Eduardo et al. (2011) reported that QTLs for external colour (EC) were located in LG 3, 6, 7 [7]. Frett (et al., 2014) detected four QTLs for skin blush: one major QTL Blush.Pp.ZC-3.1 on LG3 and three minor QTLs on LG 4 and 7 (Blush.Pp.ZC-4.1; Blush.Pp.ZC-4.2; Blush.Pp.ZC-7.1) [47]. The most significant QTLs were localised in LG3 for skin and flesh colour of apricot (García-Gómez et al., 2019) [16]. In our study, QTLs for PSC were identified in LG 1, 3, 4, and 6. The region of QTL qP-PSC3.1^1^ (Pp03: 10,648,307.. 10,838,614) overlapped with the major QTL for blush, Blush.Pp.ZC-3.1 on LG3 of the ZC^2^ SNP linkage map, residing on scaffold 3: 4,821,129..13,891,040. However, the position of QTL qP-PSC4.1^1^ (Pp04: 19,804,890.. 19,842,35) was different from the minor QTLs *Blush.Pp.ZC-4.1* (scaffold _ 4: 2,337,191..3,966,620) and Blush.Pp.ZC-4.2 (scaffold _ 4: 4,306,550..5,226,293) [47]. In addition, in this study, the red colour in flesh (RF) and red colour around pit (RP) were localized in the regions of Pp01: 24,397,147.. 26,479,742 and Pp01: 26,671,055.. 27,176,678 of LG1, respectively. Yamamoto et al. (2005) reported that flesh colour around the stone was mapped in the middle of LG 3 [18]. Cao et al. (2016) found that the SNP associated with flesh colour around the stone was located on scaffold_6: 2,183,867, scaffold_8: 16,905,885, scaffold_8: 16,795,565, scaffold_1: 31,040,363, and scaffold_1:45,251,328 [45]. These findings suggested that differences in anthocyanin content in peach flesh may be related to multiple genes.

Fruit flavour was important to consumers and thus was an important target for developing peach cultivars. Soluble sugars and acids are important components of fruit flavour. In our study, QTLs for FV were identified on LG 1, 3, 4, and 5. The region of QTL qP-FV1.2^2^ (Pp01: 27,653,862.. 28,166,262) was close to the marker BPPCT020 (Pp01:34,255,110..34,255,594), which was linked to the glucose, sorbitol, total sugar, and soluble solids content [48]. qP-FV4.1^1^ (Pp04: 15,270,537.. 17,635,472) overlapped with SSC-2007-14 (Pp04: 17, 057,020) [45]. The regions of QTLs qP-FV5.1^1^ (Pp05: 638,231.. 638,231) and qP-FV5.2^1^ (Pp05: 1,499,828.. 1499,831) overlapped with the reported positions for the D locus of acid and non-acid fruit (scaffold_5: 467,067..2,270,122) [32]. Fresnedo-Ramı’rez et al. (2015) and Boudehri et al. (2009) also found that the locus of G5Flav was associated with the D locus [42, 49]. Furthermore, QTL qP-FV5.1^1^ (Pp05: 638,231.. 638,231) was also very close to SNP_IGA_544640 (scaffold_5: 629,641), which showed the strongest association with fruit titratable acidity [50].

QTLs for SSC have previously been reported to be linked to peach LG1, 2, 4, 5, 6 [10, 12, 38, 46, 51, 52]. In apricot, García-Gómez et al. (2019) located the most significant QTLs for SSC in LG4 [16]. Our results showed that QTLs for SSC on LG1, 4, 5, and 6. On LG1, the regions of QTLs for SSC (Pp01:13,177,641.. 15,291,518 and Pp01: 24,241,572.. 24,284,070) were very close to the reported positions for SSC-2007-1 (Pp01: 15,691,351) and SSC-2007-4 (Pp01: 24,175,076), respectively [45]. However, in other linkage groups, the QTL positions of the SSCs we detected are different from those of other researchers, such as qSSC. V-Ch4–2010 was located in scaffold_4: 17,988,261 [38], QTLSSC-LG5 was located in Pp05:12,106,999..18,240,259 [49], and qSSC.6 was located between the markers ss_629062 (7,918,349) and ss_630302 (12,571,791) on LG6 [42].

For the acid content of fruit, the D major gene controlling the ‘non-acid’ fruit character was located in LG 5 (scaffold_5: 467,067..2,270,122) [32]. Moreover, QTLs for pH and titratable acidity were located near the D gene [10]. We also detected QTL loci for fruit acidity content (FA) on LG5 (Pp05: 629,308.. 677,913 and Pp05: 846,656. 1,140,022, which was consistent with the D locus. In addition, we also detected QTLs for FA on LG1, 4, and 6. Eduardo et al. (2011) reported that QTLs for FA and fruit pH were located on LG4 [7]. The region of QTL for FA we detected on LG6 was close to qTA6.1 (7,550,351.. 8,127,200) and qTA6.2(23,319,780.. 26,118,990) [18].

FDP had a non-normal distribution in progeny according to the Kruskal–Wallis test. QTLs for FDP were detected on LG 1, 2, 3, 4, 5, and 6 [12, 40, 53]. Pirona et al. (2013) located the major QTL qMD4.1 between the markers M12a (Pp04:9,219,594) and BPPCT023 (Pp04: 14,731,772) [53]. We detected 5 QTLs for FDP, of which two were located on LG 7 and three on LG 4. qP-FDF4.3^1^ (Pp04: 16,420,904) was near qMD4.1.

In summary, although we also identified some new QTLs, most of the mapping results of peach fruit quality traits overlapped or were similar to those of previous studies, which suggests that these genomic regions have important controlling roles on fruit quality traits. These stable QTLs in different genetic populations could be priorities for fine mapping, candidate gene identification and marker-assisted selection (MAS) to improve peach fruit quality.

### Candidate genes involved in fruit-related traits in peach

Candidate genes for fruit-related traits were screened and identified by phenotype-related quantitative trait loci (QTLs). Eduardo et al. (2013) identified candidate genes encoding two putative terpene synthases and one lipoxygenase (Lox), which are involved in the biosynthesis of linalool and p-menth-1-en-9-al, and nonanal, respectively [14]. Pirona et al. (2013) *identified NAC* (ppa008301m) as a candidate gene controlling maturity date in peach using QTL analysis [53]. *PpYUC11* and Prupe.6G150900.1 were identified as candidate genes for controlling the stony hard phenotype in peach [9, 54]. Nuñez-Lillo et al. (2015) screened five and nine candidate genes for maturity date and mealiness from QTL regions, respectively [3]. Cao et al. (2016; 2019) identified a large number of candidate genes controlling agronomic traits according to a genome-wide association study in peach [45, 55]. Nuñez-Lillo et al. (2019) identified candidate genes for soluble solid content, maturity date, and mealiness in peach [56]. Carrasco-Valenzuela et al. (2019) identified auxin biosynthetic pathway related genes involved in fruit softening rate by integrating conventional QTL and expression QTL (eQTL) [17]. In apricot, the candidate genes (ppa001122m, ppa000854m and ppb001660m) for the soluble solid were identified which were involved in diglucose and D-mannose binding, and transcription factor *MYB10* was found was the best candidate gene for skin colour [16].

In our study, 542 annotated genes were identified for SSC, and these annotated genes included 22 transcription factors and some softening-related cell wall remodelling degradation genes, such as pectin lyase-like superfamily proteins, polygalacturonase, expansin and xyloglucosyl transferase. Etienne et al. (2002) showed the relationship between sugar accumulation and softening processes in fruit development [51]. However, among these candidate genes, none of these genes was annotated as being involved in sugar transport or metabolism. Cao et al. (2016) also showed similar results [45]. However, among the candidate genes for FA, a large number of genes were annotated as being involved in such processes as sugar and acid synthesis, metabolism and transport, such as UDP-glucose 6-dehydrogenase (Prupe.1G284300), glucose-6-phosphate dehydrogenase (Prupe.6G307600), fructose-2,6-bisphosphatase (Prupe.6G053800), fructokinase-like (Prupe.1G289300), sucrose transporter (Prupe.1G271500), sugar transporter protein (Prupe.2G024100), V-ATPases (Prupe.4G225100), and vacuolar proton ATPase (Prupe.6G092300). UDP-glucose 6-dehydrogenase participates in the metabolic starch and sucrose metabolism pathways [57]. Glucose-6-phosphate dehydrogenase (G6PDH) has been shown to control the non-reversible dehydrogenation of glucose-6-phosphate concomitant with the reduction of NADP to NADPH [58]. The qPCR results showed that the expression of Prupe.1G284300 and Prupe.6G307600 in the parents and hybrids was positively correlated with SSC and negatively correlated with FA in the mature stages of fruits. These data suggested that Prupe.1G284300 and Prupe.6G307600 may be involved in both sugar and acid formation of peach fruits. However, further study is warranted to determine the exact function of candidate genes in the formation of SSC and FA traits in peach fruit.

By SLAF-based SNP markers, we constructed the higher density genetic maps, and identified a large number of QTLs and candidate genes for fruit quality traits, especially in terms of fruit flavor and eating quality. Fruit taste perception is not only affected by the SSC and acid content. In our study, QTLs for fruit flavour co-located with large amounts of sugar and acidity related traits, and involved in the processes as carbon metabolism, citrate cycle, and fructose and mannose metabolism, and starch and sucrose metabolism. The evaluation of intrinsic quality may provide original data for a comprehensive evaluation of fruit quality as related to its commercial potential [2]. These results are of interest to efforts to better understand the genetic mechanism of intrinsic peach quality, and also can be used to design appropriate breeding strategies to improve the intrinsic quality of commercial peach cultivars, which is the major importance for consumer.

## Conclusion

In this study, we constructed a high-density genetic map in peach based on the SLAF-seq method. This map spanned 1098.79 cM with an average distance of 0.17 cM between adjacent markers, and 90 QTLs for fruit quality related traits were mapped. From the corresponding genomic regions of these QTLs, a large number of candidate genes controlling fruit quality traits were identified. The candidate genes for fruit flavor are focused on the metabolisms and transportion of citrate, fructose, mannose, and sucrose. Candidate genes for eating quality are enriched in the synthesis and metabolisms of starch, sucrose. For SSC, the candidate genes are involved in the metabolisms of fructose, mannose, and fatty acid. For FA, the candidate genes are related to the metabolisms of starch, sucrose, and C5-branched dibasic acid. According to the qPCR, Prupe.1G284300 and Prupe.6G307600 may be involved in sugar and/or acid trait formation of peach fruits. To improve the selection efficiency for peach fruit-quality traits, the corresponding molecular markers can be developed by CAPS and/or dCAPS methods based on the genomic sequences of important candidate genes. In summary, the high density genetic map, QTLs and candidate genes studied here provide useful information for marker-assisted breeding (MAS) of peach fruit-related traits, and these results establish a foundation for the further QTL analysis, map-based cloning and functional research.

## Methods

### Plant material

An F_1_ peach population of 202 individuals derived from the cross between *Prunus persica* cv. ‘Shahong’ (SH) and *Prunus persica* cv. ‘Hongfurong’ (HFR) was used. SH was identified in 1999 by the Crop Variety Examination Committee of Shaanxi Province, China. HFR was identified in 2000 by Beijing Crop Variety Examination Committee, China. Our research team introduced SH and HFR from the National Fruit Tree Germplasm Repository, Zhengzhou Fruit Research Institute, Chinese Academy of Agricultural Sciences, in China in 2003. SH was a bud mutation of the ‘Kurakato Wase’ characterised by a early ripening time, peach with medium fruit weight, fruit skin partly coloured, medium sugar content and acidity and clingstone. HFR had been developed from a cross between ‘Qiuyu’ and ‘Xiufeng’ characterised by a late ripening time, nectarine with medium fruit weight, fruit skin partly coloured, high sugar content, low acidity and semi-freestone. Two hundred two seedlings of F_1_ offspring were grown in a nursery in 2008 and planted in the next spring on their own roots (4 × 1.0 m) in a field at the Peach Experimental Demonstration Station of Northwest A&F University (33°59’N, 107°39’E), Shaanxi Province, China. Parents and hybrids were grown under natural rainfall conditions with no irrigation, and NPK fertilizer was applied every spring. Pruning was performed yearly, and pests and diseases were controlled by conventional techniques. Hand thinning was carried out before pit hardening to a load of 60–90 fruits per tree. Fifteen fruits per tree were harvested at commercial maturity based on visual colour change and the index of absorbance difference (IAD). Fruits with an IAD between 0.8 and 1.5 were selected [59]. In addition, within the F_1_ progenies, 12 genotypes (22–10, 22–11, 23–6, 23–8, 24–5, 24–16, 24–25, 25–8, 25–14, 25–15, 25–37 and 25–38) were selected for qPCR analysis. These genotypes were selected because 24–5, 24–16 and 25–15 had high soluble solid content (SSC), while 22–10, 22–11, and 25–37 had low SSC; 23–6, 24–25 and 25–8 had high fruit acidity content (FA), while 23–8, 25–14 and 25–38 had low FA.

### DNA extraction, SLAF library construction and sequencing

Genomic DNA of parents and progenies was extracted from young leaves using the plant genomics DNA kit (Tiangen, Beijing, China) following the manufacturer’s protocol. The concentration and quality of DNA were examined by electrophoresis in 1% agarose gels and an ND-1000 spectrophotometer (NanoDrop, Wilmington, DE, USA). The SLAF-seq strategy of high-throughput sequencing was used for library construction. Briefly, the reference genome of *Prunus persica* L*.* (http://www.ncbi.nlm.nih.gov/genome/388) was used to select restriction enzyme combinations. *Rsa*I and *Hae*III (New England Biolabs, NEB, USA) were applied to digest the genomic DNA from each sample. The digested fragments were subjected to the addition of single-nucleotide A at their 3′-ends. The ligation of sequencing adapters, PCR amplification, and purification and sequencing of PCR products followed the manufacturer’s recommendations, in which the PCR fragments ranging from 264 to 364 bp were purified, and the sequencing was performed using an Illumina HiSeq™ 2500 system (Illumina Inc., San Diego, CA, USA).

### SLAF-seq data analysis and genotyping

The identification and genotyping of SLAF markers was carried out according to the method of Zhang et al. [27, 60]. Briefly, after filtering out the low-quality reads (quality score < 20e), the remaining reads were sorted to each progeny based on duplex barcode sequences. The SOAP software was used to map the clean reads with terminal 5 bp trimmed onto the peach reference genome [61]. The threshold for definition of a SLAF locus was over 95% sequence identity, and alleles in each SLAF locus were defined by the minor allele frequency evaluation. Single nucleotide polymorphisms (SNPs) were detected between parents using the software GATK (https://software.broadinstitute.org/gatk/best-practices/#variant-disco) and BWA (http://bio-bwa.sourceforge.net/), and SLAFs with > three SNPs were removed. SLAFs with more than four alleles were defined as repetitive SLAFs and discarded.

All polymorphic SLAF loci were genotyped according to the parental and offspring SNP loci. The analysis of the marker code of polymorphic SLAFs was carried out based on the software HighMap with a cross-pollinator population type (a cross between two heterozygous diploid parents), which was composed of five segregation types (ab × cd, ef × eg, hk × hk, lm × ll and nn × np). However, only three segregation types (lm × ll, nn × np and hk × hk) were genotyped in this paper. To ensure the quality of the genetic map, the valid loci for genetic mapping were filtered using the following rules. First, the lower depth genotype was set as missing, and those with more than 10 missing data points at each locus were eliminated. Second, a chi-square test was performed, and the threshold *P*-value was set to 0.01. The ‘lm × ll’ and ‘nn × np’ types had segregation ratios of 1:1, while that of ‘hk × hk’ was 1:2:1. Finally, SNPs with less than 70% integrity and parental markers that were not homologous for polymorphisms were treated the same way.

### Phenotypic data of fruit-related traits

Phenotypic identification for fruit quality characteristics in peach was performed in 2015 and 2016 according to the method of Frett et al. (2012) and Wang et al. (2005) [62, 63]. The fruit development period (FDP) was the number of days from full bloom to fruit ripening. Fruit weight (FW) and fruit diameter (FD) were measured as the average of 10 random fruit samples from each tree. The percentage of red skin colour (PSC) was a visual estimation of the surface covered. Red in flesh (RF) and red around pit (RP) were determined separately by visual estimation of the presence of red in flesh and around pit, which was scored as present 1 or absent 0 (Table 1). Flesh adherence to pit (AP) was recorded as freestone (flesh and pit completely separate), semi-freestone (flesh partially separates from pit) and clingstone (no separation between flesh and pit) (Table 3). Eating quality (EQ), fruit flavour (FV) and fruit fibre content (FFC) were determined by tasting estimation of nine breeders together with at least ten ripe fruits from each tree. EQ and FV were measured qualitatively on a scale from 1 to 5, and FFC was measured from 1 to 3 (Table 3).

**Table 3:** The methods of phenotype standardized for peach fruit quality traits

Trait	Unit of measure
Fruit development period (FDP)	days from full bloom to fruit ripening (d)
Fruit weight (FW)	grams
Fruit diameter (FD)	the diameter across cheek area (mm)
Percentage of red skin color (PSC)	%
Red in flesh (RF)	0 = no red overlay; 1 = red overlay
Red around pit (RP)	0 = no red; 1 = red
Adherence to pit (AP)	1 = freestone; 2 = semi-freestone; 3 = clingstone
Eating quality (EQ)	1 = extremely poor; 2 = poor; 3 = fair; 4 = good; 5 = excellent
Fruit flavor (FV)	1 = sour; 2 = sour-sweet; 3 = water sweet; 4 = sweet; 5 = rich sweet
Fruit fiber content (FFC)	1 = few; 2 = intermediate; 3 = many
Soluble solid content (SSC)	%
Fruit acidity content (FA)	%

Fruit samples for measuring soluble solid content (SSC, average brix degrees) and fruit acidity content (FA) were also collected at commercial maturity and were stored at 25 ± 0.5 °C with a relative humidity of 75–85%. The SSC and FA of the fruit (at least 5 fruits each time) were measured at intervals of one day during storage until the average firmness was less than 1 kg/cm^2^. SSC was measured using a refractometer (ATAGO, model PAL-1), and FA was measured using a fruit acidity meter (Korea, model GMK-835F). The methods of phenotype standardization for 12 fruit quality traits are shown in Table 3.

### Linkage map construction and QTL analysis

HighMap software (http://highmap.biomarker.com.cn/.) was used for linkage map construction [64]. The SLAF markers were mapped to the peach reference genome based on locations and then partitioned into eight linkage groups (LGs). The modified logarithm of odds (MLOD) scores between markers were calculated, and the SLAF markers that scored less than 5.0 were eliminated. The genetic distance in centimorgans (cM) was calculated using Kosambi’s mapping function. Quality assessment of the linkage map in terms of collinearity analysis. MapChart 2.3 (https://www.wur.nl/en/show/Mapchart-2.30.htm) was used to make linkage group figures.

MapQTL6.0 software (https://www.kyazma.nl/index.php/mc.MapQTL/sc.Evaluate/) was used for QTL mapping. The Kruskal–Wallis test was used to detect candidate QTLs (*P* value < 0.01). Additionally, QTLs with LOD scores greater than the threshold at a 0.99 confidence level based on a 1000-permutation test were declared significant. Neighbouring associated loci having the highest LOD scores (*P* < 0.02) were selected as co-factors in the multiple QTL model analysis.

### Identification of candidate genes

Mapping-associated markers were used to identify the homologous regions of QTLs on the physical map. Corresponding genes in QTLs were referred to the peach genome from GDR [65]. The corresponding genes in QTLs for each trait were mapped to the KEGG database (fttp://fttp.genome.jp/pub/kegg/pathway) for KEGG pathway enrichment analyses. KEGG terms with corrected *P* values < 0.05 were considered to be significantly enriched.

### RNA extraction and gene expression analysis using real-time quantitative PCR (qPCR)

Total RNA was isolated from the peach flesh using a modified PowerPlant® RNA Isolation Kit. RNA quality and integrity were detected by ultraviolet spectrophotometer and agarose gel electrophoresis. The PrimeScript RT Reagent Kit gDNA Eraser (Takara, Beijing, China) was used for converting total RNA to cDNA.

The primer sequences for qPCR were designed by Beacon Designer 8.0 (Table S3). qPCR was carried out with an iQ5 real-time PCR system (BioRad, Plano, TX, USA). The PCR was completed in a 10 μl volume containing 1 μl cDNA, 1 μl of each primer, 2 μl ddH_2_O and 5 μl SYBR Premix Ex Taq II (2×) (Takara). The qPCR programme was as follows: 1 min at 95 °C, followed by 40 cycles of 15 s at 95 °C, 20 s at 60 °C and 20 s at 72 °C. The mixed sample was heated to 95 °C for 10 s and cooled to 65 °C for 15 s. The sample was then heated to 95 °C at a rate of 0.1 °C/s for melting curve analyses. Peach 18S ribosomal RNA (18S rRNA) was used as the reference gene. Relative expression levels were analysed using the 2^−ΔΔCt^ method. Each sample was analysed in triplicate.

## Supplementary information

**Additional file 1 Figure S1**. Genetic map constructed by SNP markers. A black bar indicates an SLAF marker. The x-axis represents linkage group number, and the y-axis indicates genetic distance (centimorgan as unit).

**Additional file 2 Figure S2**. Collinearity analysis of mapping marker locations on the genetic map and peach genome. The x-axis indicates the genetic distance of each peach LG, and the y-axis represents the physical length of the LG. Markers on the map are plotted as dots.

**Additional file 3 Figure S3**. QTL location of fruit-related traits. Linkage groups, genetic distances (in centimorgans) and marker names are shown, respectively, on the top, left and right of each linkage group. QTLs are drawn by mapchart software with different RGB colours, and different traits are identified by different colours. QTLs are represented by block vertical bars positioned at the right of each linkage group. Thin lines correspond to LOD-2, and black bars correspond to the LOD-1 confidence interval.

**Additional file 4 Fig. S4** KEGG pathway enrichment analysis for candidate genes of fruit soluble solid content.

**Additional file 5 Fig. S5** KEGG pathway enrichment analysis for candidate genes of fruit acidity content.

**Additional file 6 Fig. S6** KEGG pathway enrichment analysis for candidate genes of fruit weight.

**Additional file 7 Fig. S7** KEGG pathway enrichment analysis for candidate genes of fruit flavour.

**Additional file 8 Fig. S8** KEGG pathway enrichment analysis for candidate genes of fruit eating quality.

**Additional file 9 Fig. S9** KEGG pathway enrichment analysis for candidate genes of red in flesh.

**Additional file 10 Table S1**. Candidate genes related to fruit soluble solid content (SSC) and acid content (FA).

**Additional file 11 Table S2**. Candidate genes related to fruit quality traits, including fruit weight, fruit diameter, fruit development period, percentage of red skin colour, fruit eating quality, fruit flavour, red in flesh, red around pit, adherence to pit and fruit fibre content.

**Additional file 12 Table S3**. List of primers used in this study.

## Data Availability

The datasets of raw sequence data during the current study are available in the Sequence Read Archive (SRA) under accession number PRJNA612130 [https://www.ncbi.nlm.nih.gov/bioproject/PRJNA612130]. All other data generated or analysed during this study are included in this published article and its supplementary information files.

## Abbreviations

FW: Fruit weight
FD: Fruit diameter
PSC: Percentage of red skin colour
EQ: Eating quality
RF: Red in flesh
RP: Red around pit
AP: Adherence to Pit
FDP: Fruit development period
FV: Fruit flavour
FFC: Fruit fibre content
SSC: Soluble solid content
FA: Fruit acid content
IM: Interval mapping
MQM: Multiple QTL models
cM: Centimorgan
LG: Linkage group
chr: Chromosome
LOD: Logarithm of odds
QTL: Quantitative trait loci
AFLP: Amplified Fragment Length Polymorphism
RAPD: Random Amplified Polymorphism DNA
RFLP: Restriction Fragment Length Polymorphism
SSR: Simple Sequence Repeat
SLAF-seq: Specific locus amplified fragment sequencing
SNP: Single nucleotide polymorphism
