# Supplementary figures and images for: Construction of a high-density SNP-based genetic map and identification of fruit-related QTLs and candidate genes in peach [Prunus persica (L.) Batsch]
Source: BMC Plant Biol. 2020 Sep 23;20:438. doi: 10.1186/s12870-020-02557-3 (PMC7510285; doi:10.1186/s12870-020-02557-3)

# Genetic Map

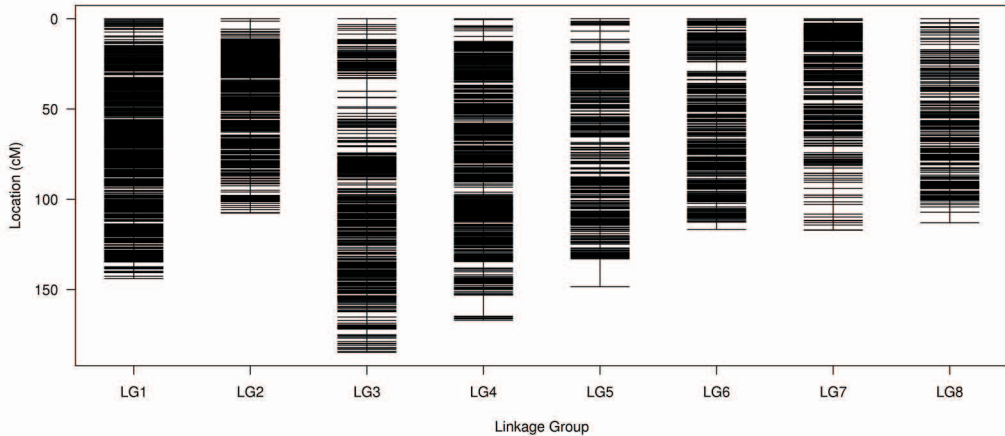

Supplement: Supplementary file 1 — Additional file 1 Figure S1. Genetic map constructed by SNP markers. A black bar indicates an SLAF marker. The x-axis represents linkage group number, and the y-axis indicates genetic distance (centimorgan as unit). [file 12870_2020_2557_MOESM1_ESM.pdf]

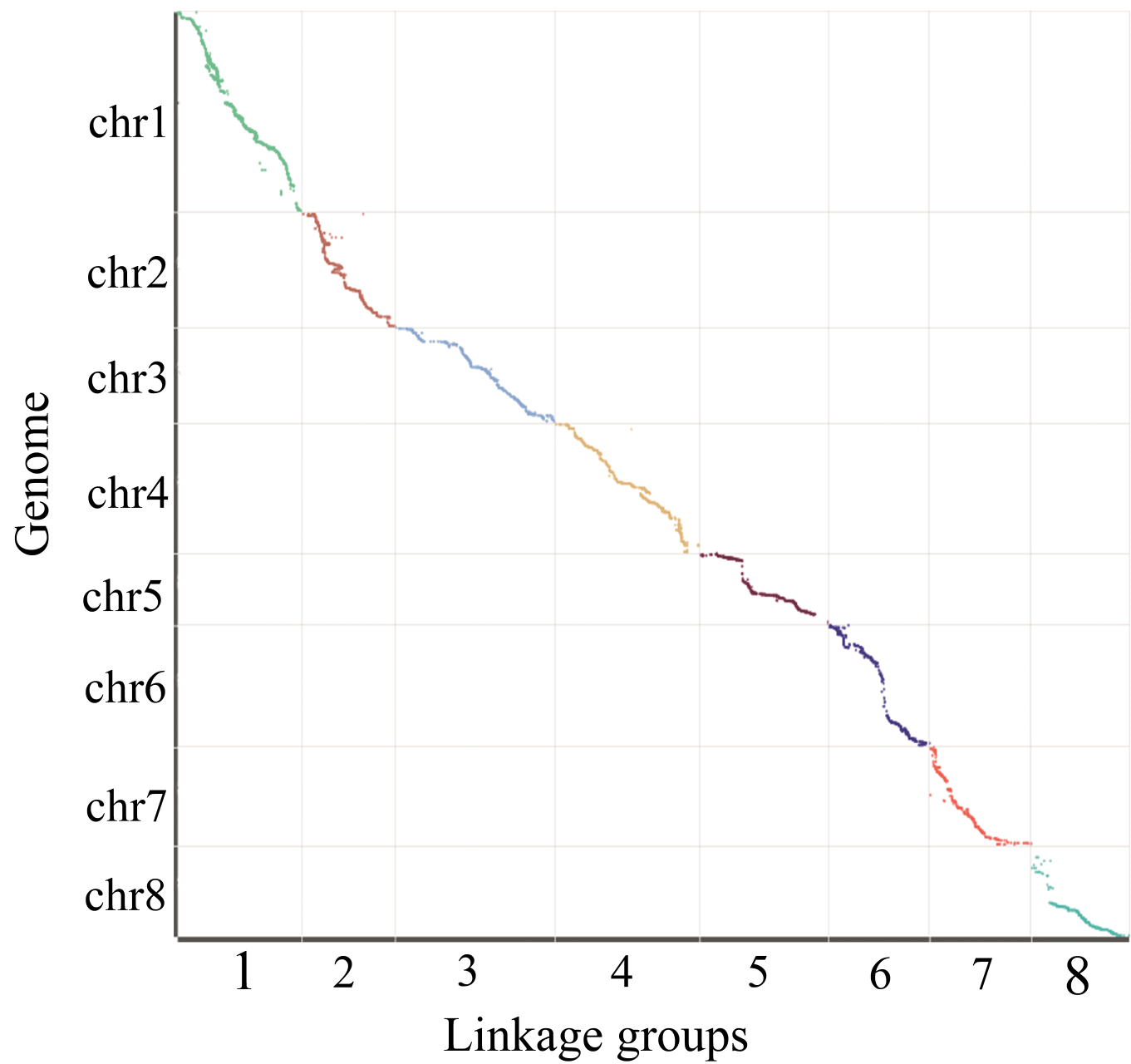

Supplement: Supplementary file 2 — Additional file 2 Figure S2. Collinearity analysis of mapping marker locations on the genetic map and peach genome. The x-axis indicates the genetic distance of each peach LG, and the y-axis represents the physical length of the LG. Markers on the map are plotted as dots. [file 12870_2020_2557_MOESM2_ESM.pdf]

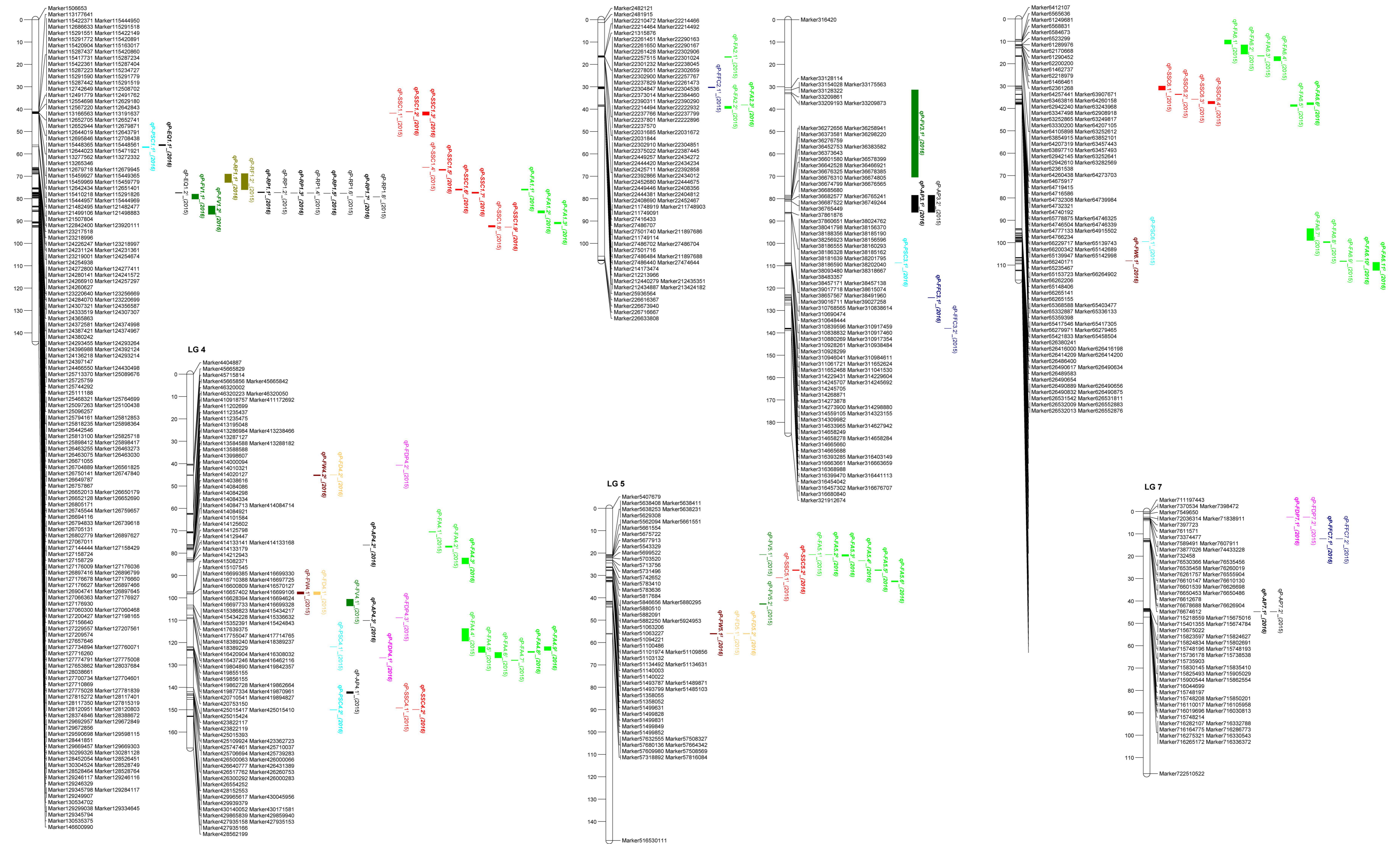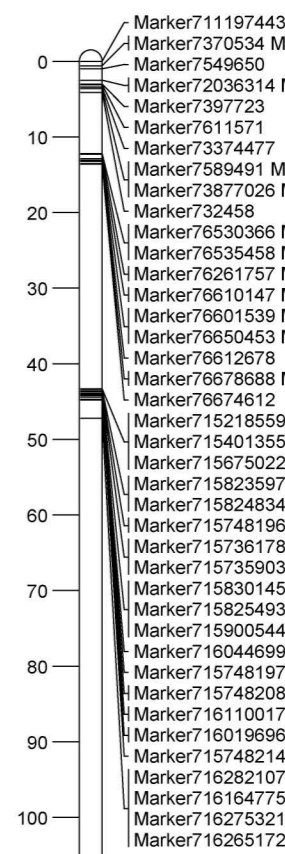

Supplement: Supplementary file 3 — Additional file 3 Figure S3. QTL location of fruit-related traits. Linkage groups, genetic distances (in centimorgans) and marker names are shown, respectively, on the top, left and right of each linkage group. QTLs are drawn by mapchart software with different RGB colours, and different traits are identified by different colours. QTLs are represented by block vertical bars positioned at the right of each linkage group. Thin lines correspond to LOD-2, and black bars correspond to the LOD-1 confidence interval. [file 12870_2020_2557_MOESM3_ESM.pdf]

## Top 20 of Pathway Enrichment

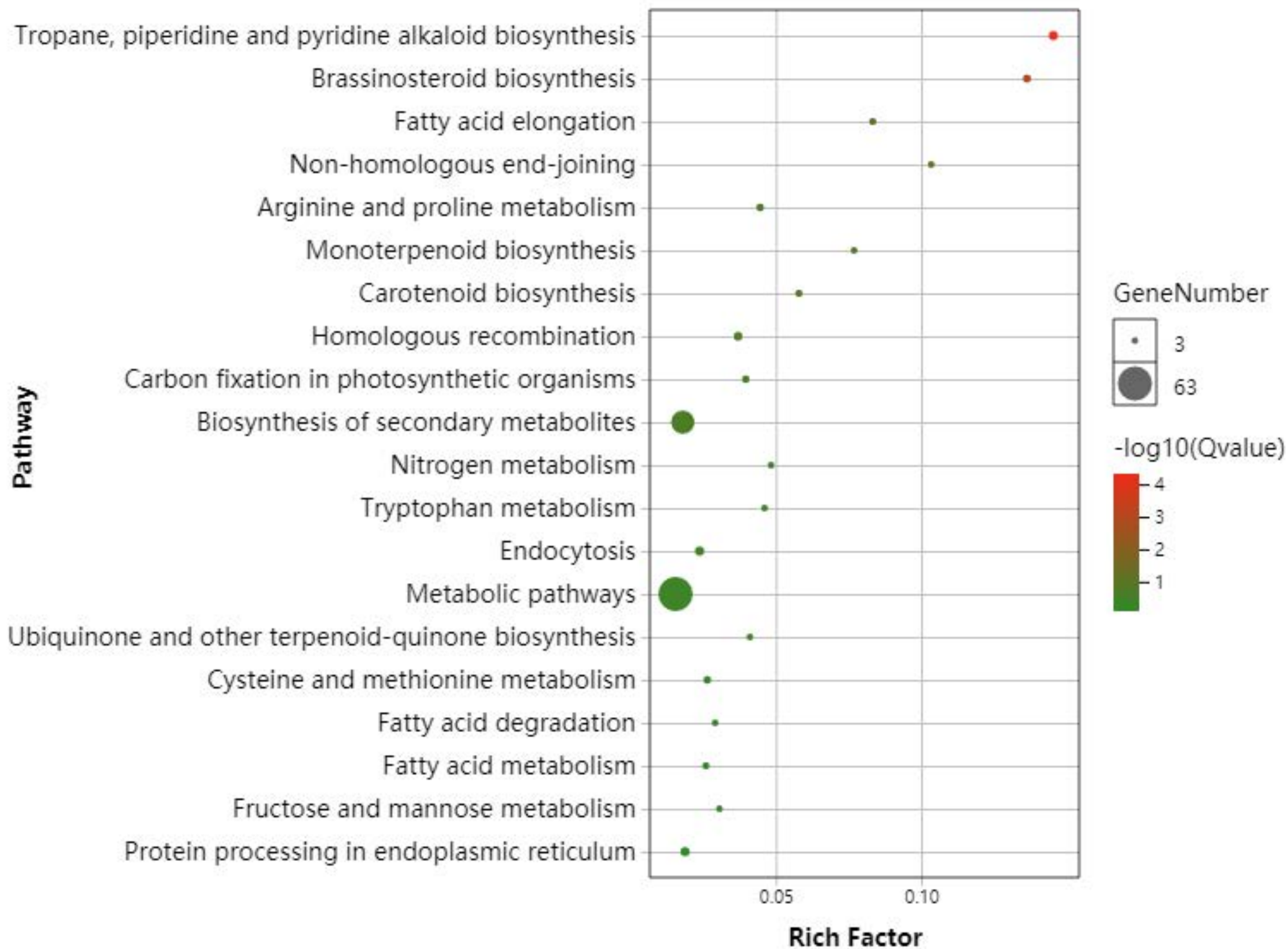

Supplement: Supplementary file 4 — Additional file 4 Fig. S4 KEGG pathway enrichment analysis for candidate genes of fruit soluble solid content. [file 12870_2020_2557_MOESM4_ESM.pdf]

## Top 20 of Pathway Enrichment

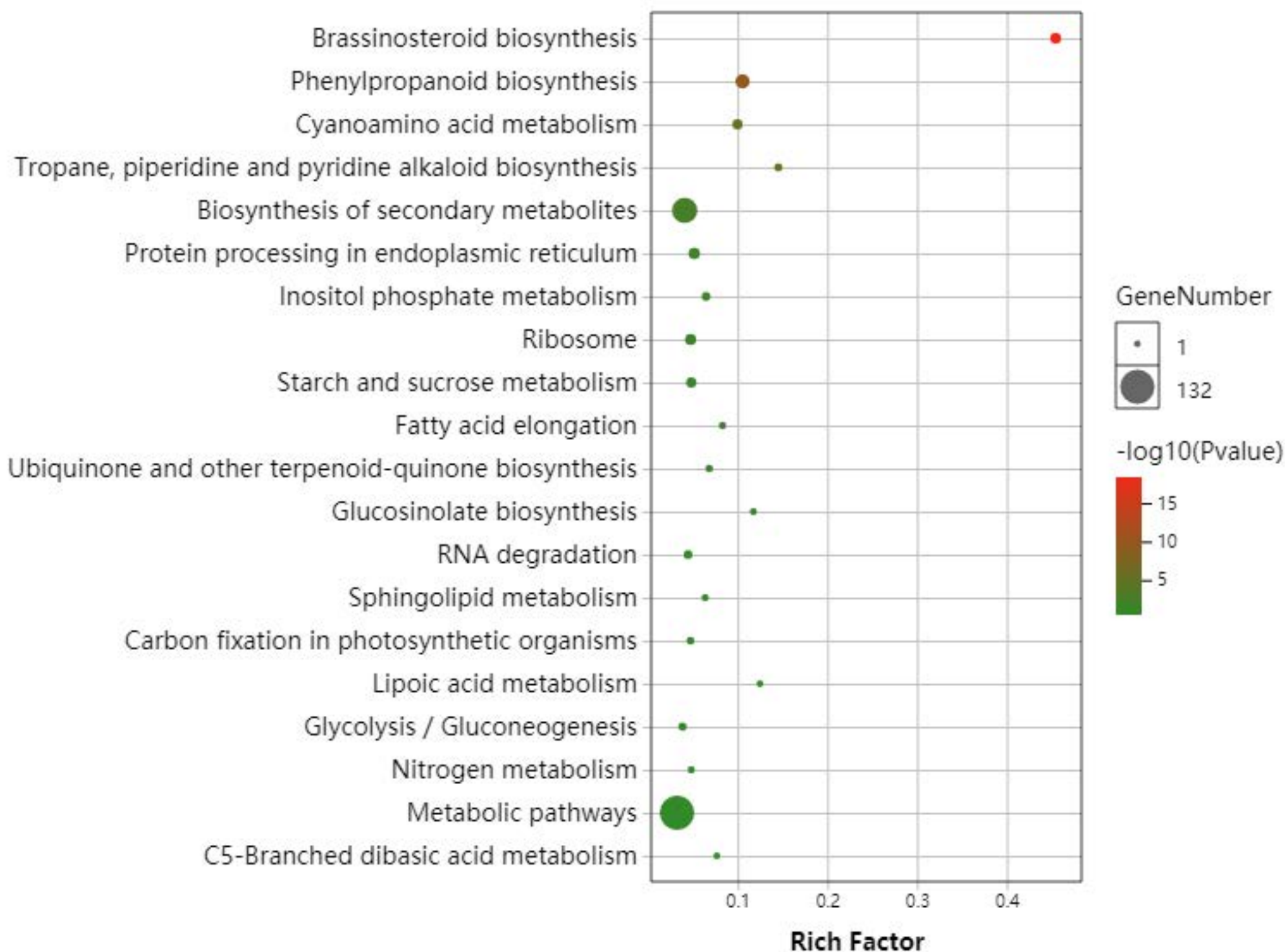

Supplement: Supplementary file 5 — Additional file 5 Fig. S5 KEGG pathway enrichment analysis for candidate genes of fruit acidity content. [file 12870_2020_2557_MOESM5_ESM.pdf]

## FW CGs of KEGG Pathway Enrichment

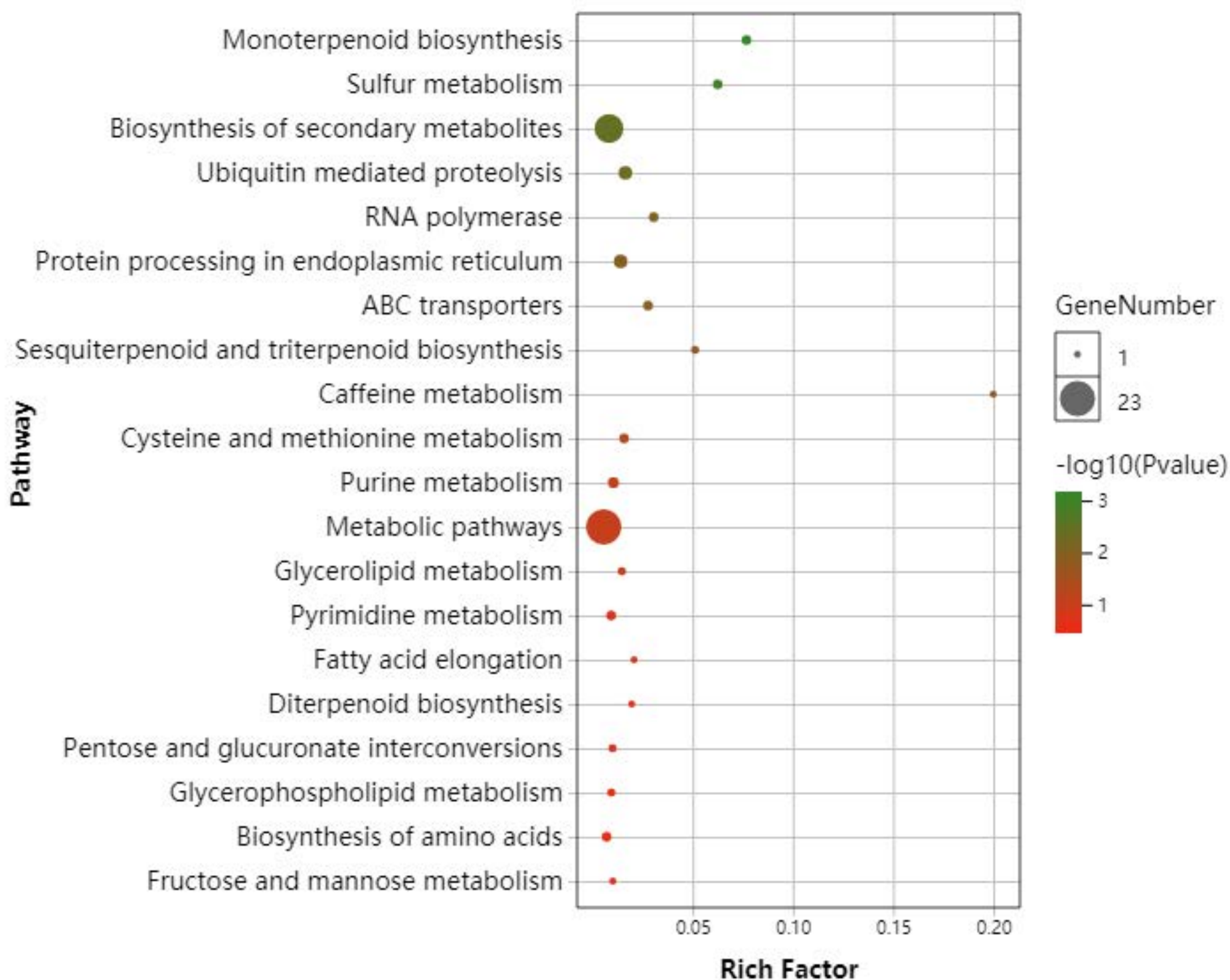

Supplement: Supplementary file 6 — Additional file 6 Fig. S6 KEGG pathway enrichment analysis for candidate genes of fruit weight. [file 12870_2020_2557_MOESM6_ESM.pdf]

# FV CGs of Pathway Enrichment

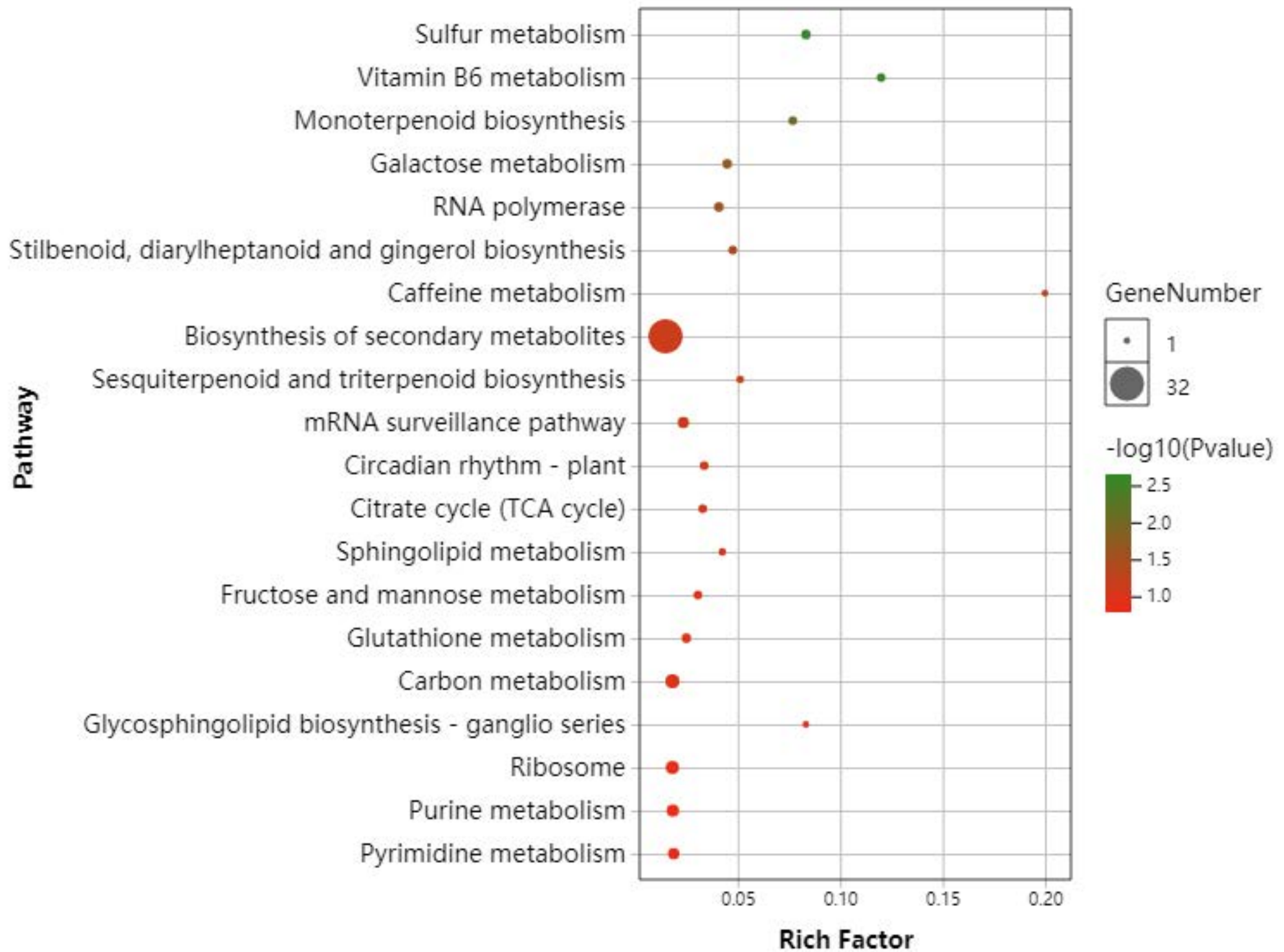

Supplement: Supplementary file 7 — Additional file 7 Fig. S7 KEGG pathway enrichment analysis for candidate genes of fruit flavour. [file 12870_2020_2557_MOESM7_ESM.pdf]

## EQ CGs of Pathway Enrichment

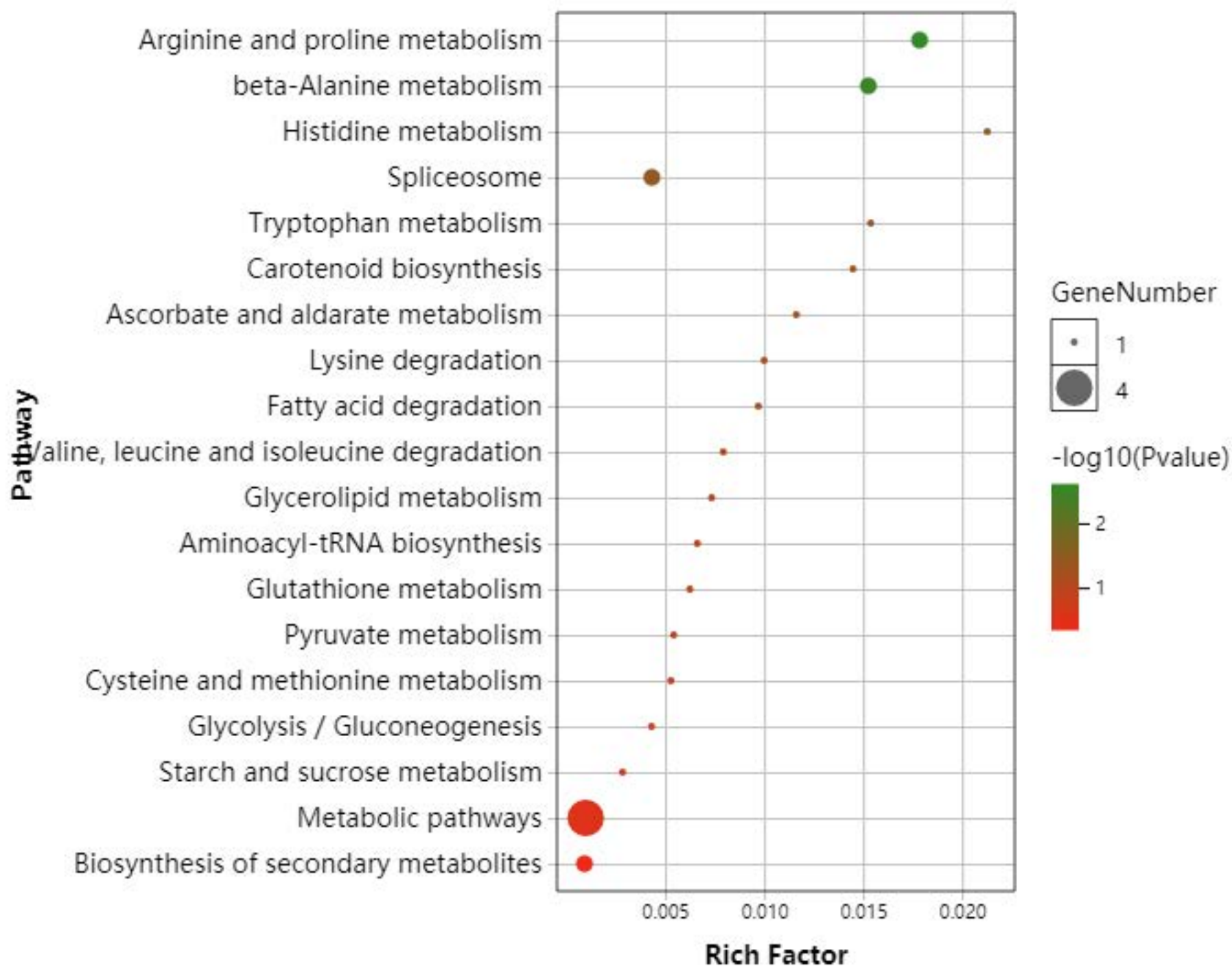

Supplement: Supplementary file 8 — Additional file 8 Fig. S8 KEGG pathway enrichment analysis for candidate genes of fruit eating quality. [file 12870_2020_2557_MOESM8_ESM.pdf]

# RF CGs of Pathway Enrichment

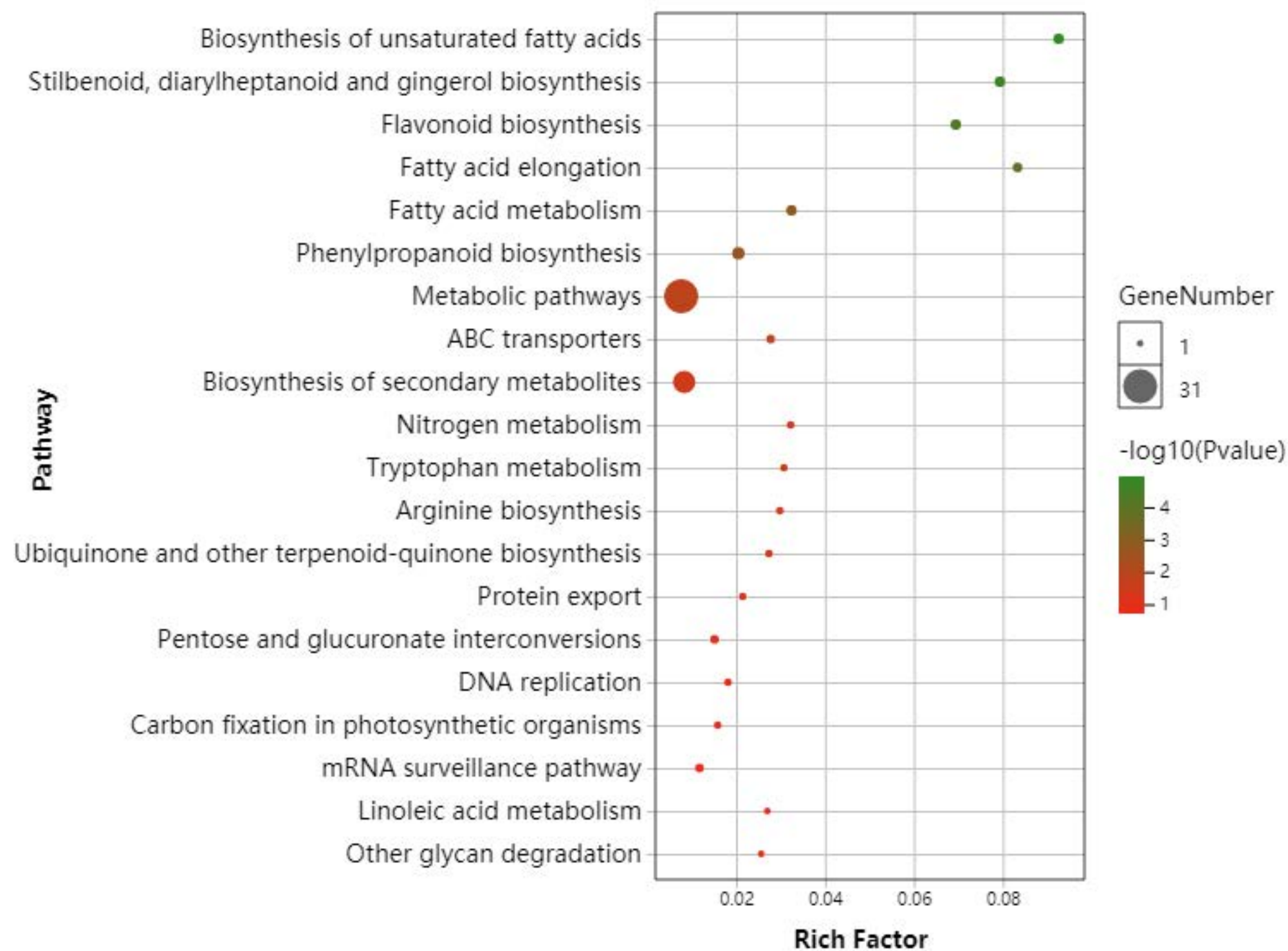

Supplement: Supplementary file 9 — Additional file 9 Fig. S9 KEGG pathway enrichment analysis for candidate genes of red in flesh. [file 12870_2020_2557_MOESM9_ESM.pdf]
